# Highly Pathogenic Avian Influenza Contributes to the Population Decline of the Peregrine Falcon (*Falco peregrinus*) in The Netherlands

**DOI:** 10.3390/v17010024

**Published:** 2024-12-27

**Authors:** Valentina Caliendo, Beatriz Bellido Martin, Ron A. M. Fouchier, Hans Verdaat, Marc Engelsma, Nancy Beerens, Roy Slaterus

**Affiliations:** 1 Dutch Wildlife Health Centre, Utrecht University, 3584 CL Utrecht, The Netherlands; 2 Department of Viroscience, Erasmus MC, 3015 GD Rotterdam, The Netherlands; a.bellidomartin@erasmusmc.nl (B.B.M.); r.fouchier@erasmusmc.nl (R.A.M.F.); 3 Wageningen Marine Research, 1781 AG Den Helder, The Netherlands; hans.verdaat@wur.nl; 4 Wageningen Bioveterinary Research, 8221 RA Lelystad, The Netherlands; marc.engelsma@wur.nl (M.E.); nancy.beerens@wur.nl (N.B.); 5 Sovon Dutch Centre for Field Ornithology, 6525 ED Nijmegen, The Netherlands; roy.slaterus@sovon.nl

**Keywords:** Peregrine Falcon, avian influenza, HPAI H5 virus

## Abstract

Highly pathogenic avian influenza (HPAI) epizootics have caused repeated mass mortality events among wild birds. The effect of the infection is potentially detrimental for a variety of bird species, including the Peregrine Falcon (*Falco peregrinus*). The numbers of wintering and breeding Peregrine Falcons in the Netherlands have recently declined. We investigated the changes in population trends in relation to HPAI H5 virus outbreaks. For this purpose, we analyzed variations in annual numbers of wintering and breeding birds, the virology of reported dead birds, and the presence of the HPAI H5 virus in unhatched eggs. We showed that significant mortalities of Peregrine Falcons had occurred in 2016–2017 and 2020–2023, years of major HPAI H5 virus outbreaks. In particular, the highest rates of bird mortality and HPAI virus infection were reported in 2023. In this year, over 80% (28/32) of the tested birds were positive for HPAI H5 virus. No HPAI H5 virus was present in the eggs. Based on these findings, we concluded that HPAI represents a serious threat to the Peregrine Falcon population in the Netherlands, and, in combination with anthropogenic factors, may contribute to the decline of this species. Targeted HPAI surveillance and disease mitigation measures are necessary for the conservation of this species.

## 1. Introduction

The latest (i.e., 2020–2023) highly pathogenic avian influenza (HPAI) H5 virus epizootics have caused repeated mass mortality events among wild birds [1,2,3]. Since 2020, the HPAI H5 virus has been detected frequently in poultry as well as in wild birds in many European countries, including the Netherlands [1,3]. The effect of the infection is potentially detrimental for the conservation of a variety of wild bird species, including the Peregrine Falcon (*Falco peregrinus*) [2]. The Peregrine Falcon is an iconic raptor species with a very large global range [4]. In the 1960s, the species declined sharply in Western Europe due to the spread of the insecticide DDT in the environment [4]. Since the ban on chlorinated hydrocarbons as insecticides and legal protection in several countries, the species has also become more common in the Netherlands. Between 1900 and 1990, the Peregrine Falcon was a very rare and incidental breeding bird in the Netherlands, but since then the species has settled in all Dutch provinces; in 2023 the Dutch population was estimated at 160–180 breeding pairs [5]. Despite this positive trend, resident population growth stalled from 2014, with significant decreases after both in 2016/2017 and 2020/2021. This also became visible in the trend of the wintering population and more recently also in the number of breeding pairs. The average number of migrating or wintering Peregrines found during monthly surveys in the winter half-year steadily increased from less than 50 individuals in the late 1990s to a maximum of circa 130 around 2013, but then dropped markedly. A similar pattern has recently become clear in the number of breeding pairs, which increased until 2021 and then started to decline too [5]. The declines correspond to times of major HPAI outbreaks in wild birds in the Netherlands. It is known that Peregrine Falcons in the Netherlands experienced increased mortality associated with HPAI in 2016/2017, 2020/2021 and 2021/2022 [1,6]. We hypothesize that mortality due to HPAI virus infection is an important cause of the recent decline of the national trends of wintering and breeding Peregrine Falcons.

We had two main objectives for our investigation: (objective 1) to quantify the consequences of the persistent presence of HPAI H5 virus on Peregrine Falcon populations in the Netherlands. Peregrine Falcons might be particularly vulnerable to HPAI H5 virus infection because of their diet, which is primarily based on other (possibly, HPAI virus-infected) bird species. Because Peregrine Falcons can consume a wide range of different bird species, we were also interested in investigating whether infection has been associated with a particular prey species. Finally, during nest monitoring activities in the spring of 2023, many abandoned nests were observed, without an obvious explanation for the findings. Therefore, we also investigated whether (objective 2) HPAI H5 virus was detected in unhatched Peregrine Falcon eggs during the spring of 2023 in the Netherlands.

## 2. Materials and Methods

A multi-level approach to the investigation:

Objective 1: Quantification of the consequences of the persistent presence of HPAI H5 virus on Peregrine Falcon populations in the Netherlands, and the possible association of infection with the consumption of a certain prey species:

- Population monitoring scheme.

Data on the distribution and population of wintering and breeding of Peregrine Falcons per year in the Netherlands were provided by the national monitoring schemes (so-called ‘Netwerk Ecologische Monitoring’) [7,8]. This monitoring and counts of live birds follow standardized guidelines and routines, and are coordinated by Sovon (Sovon) in collaboration with national and regional governmental organizations and Statistics Netherlands (CBS, Statistics Netherlands|CBS; for trend analysis and quality control).

- Wild bird mortality monitoring scheme.

Data on wild bird mortality were provided by the AI-Impact group [1]. Whitin the general monitoring of wild bird mortality, targeted surveillance for Peregrine Falcons found dead was put into place to estimate the number of HPAI virus infections and mortality rates for this species. In particular, the numbers of reported dead Peregrine Falcons during 2020–2023 on the web platforms of the AI-Impact members (https://dwhc.nl/dode-vogels-melden/, https://www.sovon.nl/nl/content/vogelgriep, https://www.nvwa.nl/onderwerpen/vogelgriep-preventie-en-bestrijding, https://waarneming.nl, URL accessed on 20 November 2024), were collected and analyzed.

- Virological testing.

We tested a limited number of Peregrine Falcons (listed in Table A1) that had been reported via the national monitoring of wild bird mortality. Oropharyngeal and cloacal swabs from these birds were tested for HPAI virus by real-time reverse transcription (RT) PCR, as previously described [9,10].

To study the relationship between viruses detected in Peregrine Falcons and other (prey) bird species, we compared full virus genome sequences of Peregrine Falcons recovered in the Netherlands between (October) 2020 and (January) 2024 using BLAST analysis in the GISAID database. We also generated a phylogenetic tree of the full virus genome sequences of Peregrine Falcons supplemented with sequences from a subset of H5 strains (15/year) selected from all Dutch strains available in the GISAID database (https://www.gisaid.org, URL accessed on 20 November 2024; Table S1). We aligned sequences by using MAFFT version 7.475 [11], reconstructed phylogeny by using maximum-likelihood analysis with IQ-TREE software version 2.0.3 [12], and visualized the maximum-likelihood tree by using the R package ggtree [13].

Objective 2: Investigation of the presence of HPAIV in unhatched Peregrine Falcon eggs during the spring of 2023 in the Netherlands:

- The presence of HPAI H5 virus in unhatched eggs.

Seventeen unhatched eggs from twelve different Peregrine Falcon nests from different locations, submitted to the Dutch Raptor Working group (“Werkgroep Roofvogels Nederland”, werkgroeproofvogels.nl) during the spring of 2023, were investigated virologically for the presence of HPAI virus, and serologically for the presence of H5 antibodies. The eggs were collected from nests at the end of the breeding season, and after no adult birds were seen attending the nests for several consecutive days. Briefly, external eggshells and egg yolks were swabbed and tested by RT-PCR [9]. Egg yolks were tested for H5 antibodies with a commercial H5 indirect ELISA (IDVet Influenza H5 indirect, ID Screen^®^), following the instructions from the manufacturer. Three of the unhatched eggs contained a well-developed embryo. Different embryonal tissues from the three embryos were homogenized and the tissues were tested in a triplex RT-qPCR targeting the matrix gene and H5 gene of influenza A virus and the HA gene of Phocine Distemper Virus (PDV) as an internal control [14]. No history of the parents was available.

## 3. Results

Objective 1: Quantification of the consequences of the persistent presence of HPAI H5 virus on Peregrine Falcon populations in the Netherlands, and possible association of infection with the consumption of a certain prey species:

- Population monitoring scheme.

**Figure 1:**
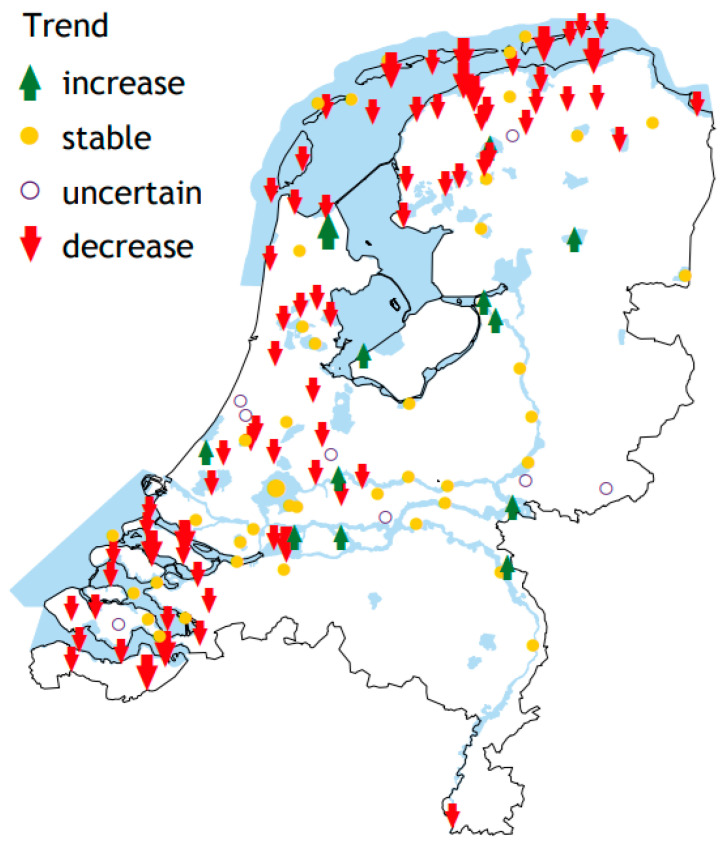
Trends of wintering Peregrine Falcons across the Netherlands in the period from 2017/2018 to 2022/2023 based on the Waterbird Monitoring Scheme (Sovon, Netwerk Ecologische Monitoring).

The average number of Peregrine Falcons from July to June of the following year (based on monthly counts throughout the year that were then averaged for a 12-month period) in the Netherlands in 2008/2009 and 2022/2023 based on the Waterbird Monitoring Scheme, with the periods in which high wild bird mortality associated with HPAI infections occurred marked in gray, is shown in Table 1. The largest decrease in terms of percentage difference compared to the previous year occurred in 2017/2018 and 2021/2022 with decreases of >15% and >22%, respectively. These declines were noted in a large part of the country, particularly in coastal areas. However, in certain areas numbers stayed stable or even slightly increased over the period from 2017/2018 to 2022/2023 (Figure 1).

The number of breeding pairs of Peregrine Falcons in the Netherlands in 2018–2023 is given in Table 2. A decline has become visible only recently; the highest decrease in terms of percentage difference compared to the previous year occurred in 2023 (−17%).

- Wild bird mortality monitoring scheme.

**Figure 2:**
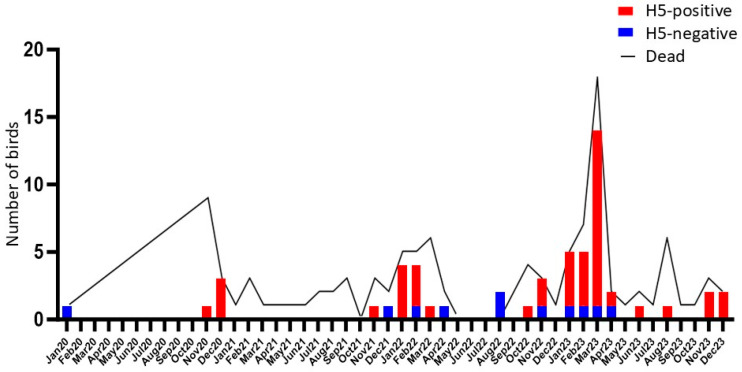
Number of Peregrine Falcons reported dead, tested, and found positive for HPAI virus per month from 2020 to 2023 in the Netherlands.

Since 2020, the yearly numbers of reported deaths and HPAI H5 virus-infected Peregrine Falcons have progressively increased (Figure 2, Table A1 and Table A2). Differences in the age and sex of the infected falcons were tested for the possibility of increased susceptibility to infection for a particular age or sex group; however, there were no statistically significant differences (ANOVA analysis, *p* > 0.1).

- Virological testing.

**Figure 3:**
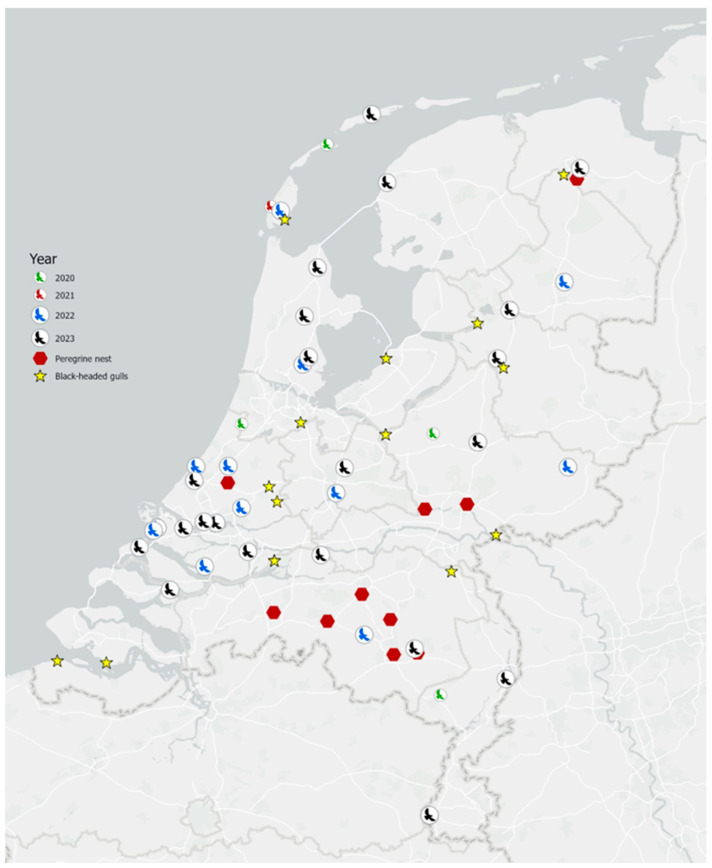
Yearly geographical distribution, from 2020 to 2023, of reported dead, H5 HPAI virus-infected Peregrine Falcons in the Netherlands. Red hexagons indicate the location of the nests tested in 2023. Yellow stars indicate the sites of large (≥30 birds) H5-related mortality events among Black-Headed Gulls in 2023.

The peak of mortalities and infections occurred during the autumn and winter months (i.e., October to February) (Figure 2). These seasons also correspond to the time of highest presence of (migratory) Peregrine Falcons in the country. Infection can; however, occur throughout the year, and in 2023 a minority of birds tested positive for HPAI H5 virus also in spring and summer. The highest rates of mortality and infection were reported during the year 2023. Geographically, infections have occurred all over the Netherlands, without noticeable clusters (Figure 3).

**Figure 4:**
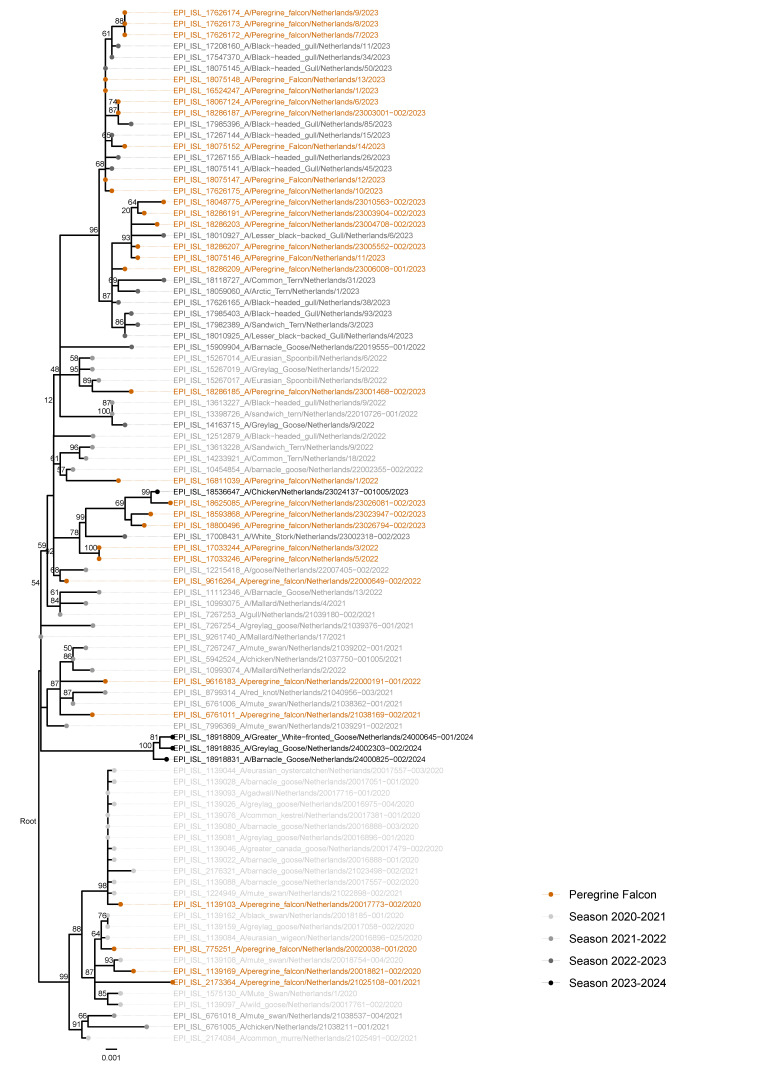
Phylogenetic tree of the HA gene of HPAI H5 viruses from Dutch Peregrine Falcons with a selection of additional HPAI strains from GISAID of other hosts in the Netherlands. Tree topology was evaluated by 1000 bootstrap analyses, the values above 50 are indicated at the branch nodes.

In the phylogenetic analysis (Figure 4), Peregrine Falcon sequences did not cluster together, but rather clustered with other wild bird sequences of the same date. In earlier years (i.e., 2020/2021), Peregrine Falcon sequences were most frequently related to AB genotype viruses from Anatidae [3]. However, in 2022/2023 sequences were more commonly of the BB genotype, the same genotype that was recovered in HPAI virus outbreaks in gulls and terns in the same years. This is in line with the dominant epidemiological patterns in the Netherlands during those years. It is therefore likely that the increased numbers of Peregrine Falcon mortality and infection in 2023 were related to the HPAI virus outbreaks in Laridae breeding colonies and roosts.

Based on the phylogenetic analysis of the HPAI H5 virus, the closest relative sequences to the sequences of the Peregrine Falcons were often not geographically close, and were often isolated from birds that were unusual prey for the Peregrine Falcon, such as Mute Swan (*Cygnus olor*) and goose species (*Branta* sp. and *Anser* sp.) (Figure 4 and Figure S1, Table S1). Though it has to be noted that scavenging occurs too in Peregrine Falcons [15]. In 2023, Peregrine Falcon sequences were closely related to Black-Headed Gull (*Chroicocephalus ridibundus*) sequences, coinciding with a major HPAI H5 virus outbreak in Black-Headed Gull populations in the Netherlands, and most likely because Peregrine Falcons were feeding on infected gulls. Sequences from Peregrine Falcons and other bird species are linked in time: they are observed at approximately the same date (+/− one month) to each other. Viral sequences of Peregrine Falcons are representative of the avian influenza strain present at that time period in the Netherlands.

Objective 2: Investigation of the presence of HPAIV in Peregrine Falcon unhatched eggs during the spring of 2023 in the Netherlands:

- The presence of HPAI H5 virus in unhatched eggs.

All samples collected from the Peregrine Falcon eggs tested negative, indicating no presence of the virus or antibodies against Influenza A virus or H5 HPAI virus.

## 4. Discussion

The Peregrine Falcon is a cosmopolitan bird of prey (raptor) in the family Falconidae. The Peregrine Falcon has successfully adapted to a man-made landscape and is now an example of urban wildlife [16]. Recently, the species had slowly recovered from several anthropogenic threats, such as the unregulated use of pesticides and poaching, when a new threat made it relapse. As is the case with other raptor species, the Peregrine Falcon is at high risk of infection by the HPAI H5 virus [17,18,19]. Being a predator, with a diet mainly composed of other birds, as well as being an opportunistic scavenger, it is exposed to important risk factors for infection.

The Peregrine Falcon is a partially migratory bird, with the northernmost populations being almost completely migratory, while more southern populations are predominantly sedentary. Birds from northern Scandinavia and northern Russia leave their breeding areas completely and migrate south, as far as Africa [4]. In the Netherlands, both locally breeding individuals as well as migratory ones (particularly from Scandinavia) occur. It is possible that the higher level of winter mortality in our study substantially affected the migratory population. Although, in our study, we found no direct evidence for this, as none of the dead birds had been ringed or tagged, declines have also been reported from Scandinavian breeding populations. Monitoring efforts in Finland have shown that the number of pairs steadily increased from the 1980s up to 2015 (207 pairs), but have declined since (124 pairs in 2024) [20]. In Sweden, a large decline was noted in 2021, when the total number of chicks raised by Peregrine Falcons was about half of that in 2020, and the population has not yet fully recovered since then [21]. In Denmark, the numbers dropped from 17 pairs in 2020 to 9 in 2023 [22].

The Dutch Peregrine Falcon wintering population showed major declines in 2017, 2022 and 2023, following large HPAI virus outbreaks in wild birds in western Europe. In 2017, the decline was stronger in the north of the country than in the southwest, in accordance with the distribution pattern of HPAI-infected waterbirds at the time [2]. Since then, declines have been noted all over the country, with an emphasis on wetlands where many HPAI virus-infected prey (i.e., medium-sized waterbirds) have been present in recent years. It is striking that there are still isolated, inland, population increases. It is likely that the Peregrine Falcons in those areas feed more on pigeons (an uncommon host for the avian influenza virus) and less on waterbirds (a common host for the influenza virus), and therefore face a lower risk of becoming infected.

The Peregrine Falcon consumes a wide range of different species, and food choice is strongly determined by supply, with the exact species composition determined by the location [23]. In rural areas, medium-sized waders, ducks, pigeons, starlings, etc., are common prey items, but at times, larger prey up to geese are also hunted. In urban areas, pigeons are preferred. Furthermore, the Peregrine Falcon is attracted by large concentrations of birds, such as waterbirds aggregated in wetlands, as well as by prey showing deviant behavior, such as HPAI virus-infected birds showing neurological signs.

The result of our virus-phylogenetic analysis show that sequences of Peregrine Falcons did not cluster together, but instead represented the sequences of their prey. Meaning that Peregrine Falcons did not infect each other, but rather they acquired the infection via their prey. Different virus lineages affected first Anseriformes (AB) and later Laridae (BB) and both lineages affected Peregrine Falcons; thus, Peregrine Falcons likely were infected from consuming both classes of birds.

Sequences were not always geographically, but more chronologically, linked. Therefore, viral sequences of Peregrine Falcons are valuable for surveillance, as they are representative of the avian influenza strain present at that time period in the country.

In the Netherlands, Peregrine Falcons breed between February and April. The female lays 2–4 eggs, which are incubated for circa 30 days. After hatching, the young falcons stay in the nest for up to 42 days, and become independent after a further 2 months [23]. As we did not find evidence of infection in the eggs that we tested, it is possible that the nest failure was indirectly due to HPAI, in the event, for example, that the parents succumbed to disease. Peregrine Falcons often hunt far from the nest, in open landscapes; it is then unlikely that the dead parents would be recovered in proximity to the nest.

In conclusion, HPAI represents a serious threat for the Peregrine Falcon in the Netherlands, and, in combination with other anthropogenic factors, can contribute to the decline of this species. As the dynamics of the HPAI H5 virus are still difficult to predict, targeted surveillance and disease mitigation measures, for example, prompt carcasses removal of infected dead prey birdss during mass mortality events, should be set in place to ensure the conservation of the Peregrine Falcon and other vulnerable species.

## Figures and Tables

**Table 1:** The average number of Peregrine Falcons from July to June of the following year in the Netherlands in 2008/2009 and 2022/2023, based on the Waterbird Monitoring Scheme, with the periods in which high wild bird mortality associated with HPAI infections occurred marked in gray. The number in 2022/2023 is a provisional estimate.

Season	Average Number	Percentage Difference Compared to Previous Year
2008/2009	102	
2009/2010	92	−9.8
2010/2011	110	18.8
2011/2012	120	8.9
2012/2013	117	−1.9
2013/2014	122	3.9
2014/2015	118	−2.9
2015/2016	121	2.3
2016/2017	113	−6.4
2017/2018	96	−15.5
2018/2019	93	−2.5
2019/2020	106	13.9
2020/2021	94	−11.5
2021/2022	73	−22.6
2022/2023	66	−9.6

**Table 2:** The number of breeding pairs of Peregrine Falcons in the Netherlands in 2018–2023, based on the Breeding Birds Monitoring Scheme, with the period in which high wild bird mortality associated with HPAI infections occurred marked in gray.

Year	Number Beeding Pairs	Percentage Difference Compared to Previous Year
2018	180–200	
2019	190–210	5
2020	190–210	0
2021	210–240	13
2022	190–220	−9
2023	160–180	−17

## Data Availability

All data are available in the article and Appendix A.

## Supplementary Materials

The following supporting information can be downloaded at: https://www.mdpi.com/article/10.3390/v17010024/s1, Figure S1: The phylogenetic trees of all gene segments of HPAI H5 viruses from Dutch Peregrine Falcons with a selection of additional HPAI strains from GISAID of other hosts in the Netherlands. The tree topology was evaluated by 1000 UltraFast bootstrap analyses (IQTree); the values are indicated at the branch nodes. Table S1: Acknowledgement of the authors and the originating and submitting laboratories of the sequences from GISAID’s EpiFlu™ Database on which this research is based.

## Appendix A

**Table A1:** The number of Peregrine Falcons reported dead, tested, and found positive for the HPAI virus per month from 2020 to 2023 in the Netherlands.

	2020			2021			2022			2023		
	Dead	Tested	H5-Positive	Dead	Tested	H5-Positive	Dead	Tested	H5-Positive	Dead	Tested	H5-Positive
Jan	1	1	0	1	0	0	5	4	4	5	5	4
Feb		0	0	3	0	0	5	2	3	7	5	4
Mar		0	0	1	0	0	6	1	1	18	14	13
Apr		0	0	1	0	0	2	1	0	2	2	1
May		0	0	1	0	0	0	0	0	1	0	0
Jun		0	0	1	0	0	0	0	0	2	1	1
Jul		0	0	2	0	0	0	0	0	1	0	0
Aug		0	0	2	0	0	0	2	0	6	1	1
Sep		0	0	3	0	0	2	0	0	1	0	0
Oct		0	0	0	0	0	4	1	1	1	0	0
Nov	9	1	1	3	1	1	3	3	2	3	2	2
Dec	3	3	3	2	1	0	1	0	0	2	2	2
Total	13	5	4	20	2	1	28	14	11	49	32	28

**Table A2:** The age and sex of Peregrine Falcons found dead and positive for HPAI virus from 2020 to 2023 in the Netherlands.

Year	2020	2021	2022	2023
Age	1 Male, 3 Unknown	1 Unknown	1 Adult, 4 Immature, 6 Unknown	10 Adut, 12 Immature, 6 Unknown
Gender	1 Adult, 3 Unknown	1 Unknown	2 Male, 4 Female, 5 Unknown	8 Male, 10 Female, 10 Unknown
