# Supplementary material for: Highly Pathogenic Avian Influenza Contributes to the Population Decline of the Peregrine Falcon (Falco peregrinus) in The Netherlands
Source: Viruses. 2024 Dec 27;17(1):24. doi: 10.3390/v17010024 (PMC11768999; doi:10.3390/v17010024)
Supplement: Supplementary file 1 [file viruses-17-00024-s001.zip › Figure S1 Phylogenetic tree of all gene segments HPAI H5 viruses from Dutch peregrine falcons.pdf]

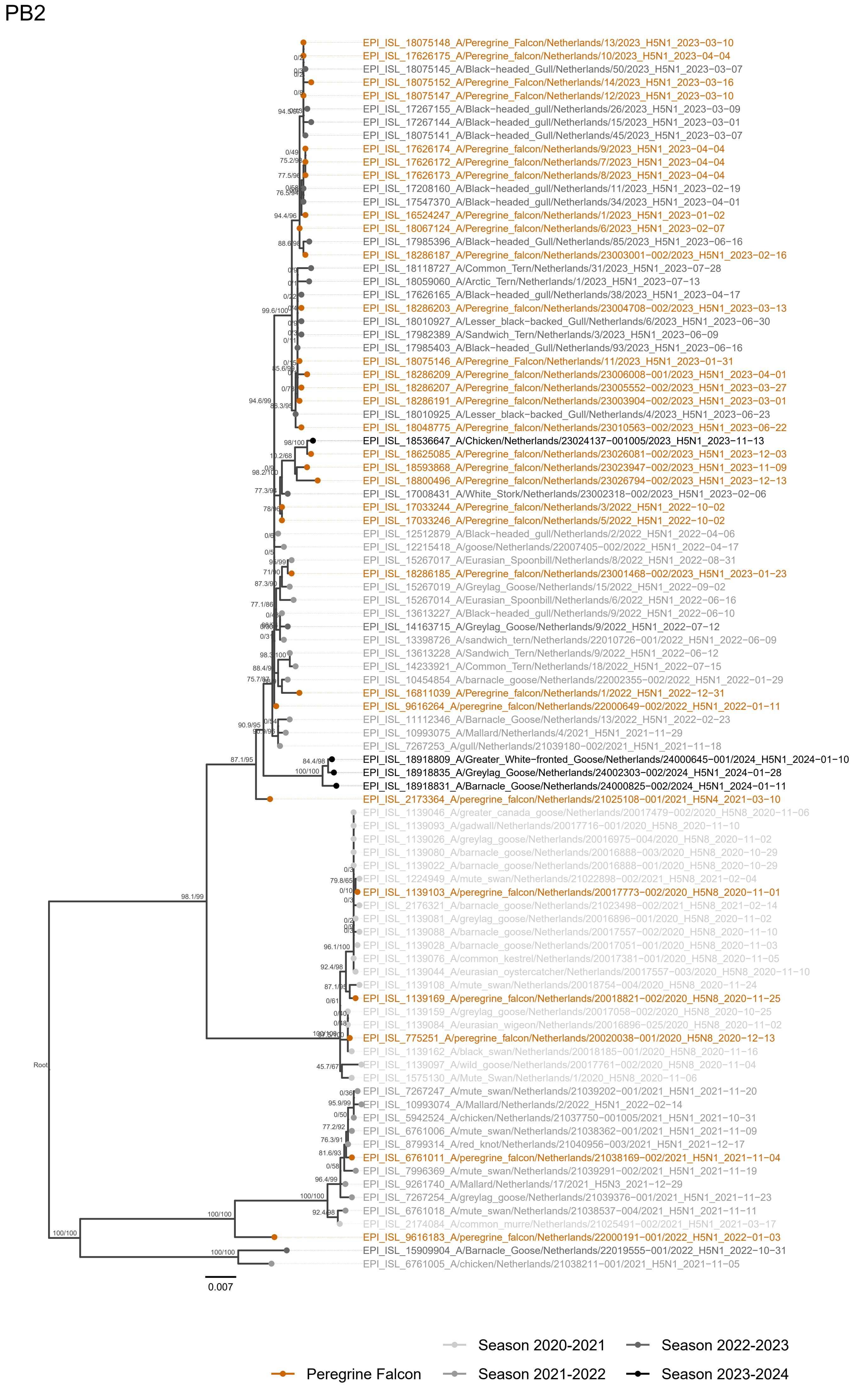
PB2
EPI_ISL_18075148_A/Peregrine_Falcon/Netherlands/13/2023_H5N1_2023−03−10
EPI_ISL_17626175_A/Peregrine_falcon/Netherlands/10/2023_H5N1_2023−04−04
EPI_ISL_18075145_A/Black−headed_Gull/Netherlands/50/2023_H5N1_2023−03−07
EPI_ISL_18075152_A/Peregrine_Falcon/Netherlands/14/2023_H5N1_2023−03−16
EPI_ISL_18075147_A/Peregrine_Falcon/Netherlands/12/2023_H5N1_2023−03−10
EPI_ISL_17267155_A/Black−headed_gull/Netherlands/26/2023_H5N1_2023−03−09
EPI_ISL_17267144_A/Black−headed_gull/Netherlands/15/2023_H5N1_2023−03−01
EPI_ISL_18075141_A/Black−headed_Gull/Netherlands/45/2023_H5N1_2023−03−07
EPI_ISL_17626174_A/Peregrine_falcon/Netherlands/9/2023_H5N1_2023−04−04
EPI_ISL_17626172_A/Peregrine_falcon/Netherlands/7/2023_H5N1_2023−04−04
EPI_ISL_17626173_A/Peregrine_falcon/Netherlands/8/2023_H5N1_2023−04−04
EPI_ISL_17208160_A/Black−headed_gull/Netherlands/11/2023_H5N1_2023−02−19
EPI_ISL_17547370_A/Black−headed_gull/Netherlands/34/2023_H5N1_2023−04−01
EPI_ISL_16524247_A/Peregrine_falcon/Netherlands/1/2023_H5N1_2023−01−02
EPI_ISL_18067124_A/Peregrine_falcon/Netherlands/6/2023_H5N1_2023−02−07
EPI_ISL_17985396_A/Black−headed_Gull/Netherlands/85/2023_H5N1_2023−06−16
EPI_ISL_18286187_A/Peregrine_falcon/Netherlands/23003001−002/2023_H5N1_2023−02−16
EPI_ISL_18118727_A/Common_Tern/Netherlands/31/2023_H5N1_2023−07−28
EPI_ISL_18059060_A/Arctic_Tern/Netherlands/1/2023_H5N1_2023−07−13
EPI_ISL_17626165_A/Black−headed_gull/Netherlands/38/2023_H5N1_2023−04−17
EPI_ISL_18286203_A/Peregrine_falcon/Netherlands/23004708−002/2023_H5N1_2023−03−13
EPI_ISL_18010927_A/Lesser_black−backed_Gull/Netherlands/6/2023_H5N1_2023−06−30
EPI_ISL_17982389_A/Sandwich_Tern/Netherlands/3/2023_H5N1_2023−06−09
EPI_ISL_17985403_A/Black−headed_Gull/Netherlands/93/2023_H5N1_2023−06−16
EPI_ISL_18075146_A/Peregrine_Falcon/Netherlands/11/2023_H5N1_2023−01−31
EPI_ISL_18286209_A/Peregrine_falcon/Netherlands/23006008−001/2023_H5N1_2023−04−01
EPI_ISL_18286207_A/Peregrine_falcon/Netherlands/23005552−002/2023_H5N1_2023−03−27
EPI_ISL_18286191_A/Peregrine_falcon/Netherlands/23003904−002/2023_H5N1_2023−03−01
EPI_ISL_18010925_A/Lesser_black−backed_Gull/Netherlands/4/2023_H5N1_2023−06−23
EPI_ISL_18048775_A/Peregrine_falcon/Netherlands/23010563−002/2023_H5N1_2023−06−22
EPI_ISL_18536647_A/Chicken/Netherlands/23024137−001005/2023_H5N1_2023−11−13
EPI_ISL_18625085_A/Peregrine_falcon/Netherlands/23026081−002/2023_H5N1_2023−12−03
EPI_ISL_18593868_A/Peregrine_falcon/Netherlands/23023947−002/2023_H5N1_2023−11−09
EPI_ISL_18800496_A/Peregrine_falcon/Netherlands/23026794−002/2023_H5N1_2023−12−13
EPI_ISL_17008431_A/White_Stork/Netherlands/23002318−002/2023_H5N1_2023−02−06
EPI_ISL_17033244_A/Peregrine_falcon/Netherlands/3/2022_H5N1_2022−10−02
EPI_ISL_17033246_A/Peregrine_falcon/Netherlands/5/2022_H5N1_2022−10−02
EPI_ISL_12512879_A/Black−headed_gull/Netherlands/2/2022_H5N1_2022−04−06
EPI_ISL_12215418_A/goose/Netherlands/22007405−002/2022_H5N1_2022−04−17
EPI_ISL_15267017_A/Eurasian_Spoonbill/Netherlands/8/2022_H5N1_2022−08−31
EPI_ISL_18286185_A/Peregrine_falcon/Netherlands/23001468−002/2023_H5N1_2023−01−23
EPI_ISL_15267019_A/Greylag_Goose/Netherlands/15/2022_H5N1_2022−09−02
EPI_ISL_15267014_A/Eurasian_Spoonbill/Netherlands/6/2022_H5N1_2022−06−16
EPI_ISL_13613227_A/Black−headed_gull/Netherlands/9/2022_H5N1_2022−06−10
EPI_ISL_14163715_A/Greylag_Goose/Netherlands/9/2022_H5N1_2022−07−12
EPI_ISL_13398726_A/sandwich_tern/Netherlands/22010726−001/2022_H5N1_2022−06−09
EPI_ISL_13613228_A/Sandwich_Tern/Netherlands/9/2022_H5N1_2022−06−12
EPI_ISL_14233921_A/Common_Tern/Netherlands/18/2022_H5N1_2022−07−15
EPI_ISL_10454854_A/barnacle_goose/Netherlands/22002355−002/2022_H5N1_2022−01−29
EPI_ISL_16811039_A/Peregrine_falcon/Netherlands/1/2022_H5N1_2022−12−31
EPI_ISL_9616264_A/peregrine_falcon/Netherlands/22000649−002/2022_H5N1_2022−01−11
EPI_ISL_11112346_A/Barnacle_Goose/Netherlands/13/2022_H5N1_2022−02−23
EPI_ISL_10993075_A/Mallard/Netherlands/4/2021_H5N1_2021−11−29
EPI_ISL_7267253_A/gull/Netherlands/21039180−002/2021_H5N1_2021−11−18
EPI_ISL_18918809_A/Greater_White−fronted_Goose/Netherlands/24000645−001/2024_H5N1_2024−01−10
EPI_ISL_18918835_A/Greylag_Goose/Netherlands/24002303−002/2024_H5N1_2024−01−28
EPI_ISL_18918831_A/Barnacle_Goose/Netherlands/24000825−002/2024_H5N1_2024−01−11
EPI_ISL_2173364_A/peregrine_falcon/Netherlands/21025108−001/2021_H5N4_2021−03−10
EPI_ISL_1139046_A/greater_canada_goose/Netherlands/20017479−002/2020_H5N8_2020−11−06
EPI_ISL_1139093_A/gadwall/Netherlands/20017716−001/2020_H5N8_2020−11−10
EPI_ISL_1139026_A/greylag_goose/Netherlands/20016975−004/2020_H5N8_2020−11−02
EPI_ISL_1139080_A/barnacle_goose/Netherlands/20016888−003/2020_H5N8_2020−10−29
EPI_ISL_1139022_A/barnacle_goose/Netherlands/20016888−001/2020_H5N8_2020−10−29
EPI_ISL_1224949_A/mute_swan/Netherlands/21022898−002/2021_H5N8_2021−02−04
EPI_ISL_1139103_A/peregrine_falcon/Netherlands/20017773−002/2020_H5N8_2020−11−01
EPI_ISL_2176321_A/barnacle_goose/Netherlands/21023498−002/2021_H5N8_2021−02−14
EPI_ISL_1139081_A/greylag_goose/Netherlands/20016896−001/2020_H5N8_2020−11−02
EPI_ISL_1139088_A/barnacle_goose/Netherlands/20017557−002/2020_H5N8_2020−11−10
EPI_ISL_1139028_A/barnacle_goose/Netherlands/20017051−001/2020_H5N8_2020−11−03
EPI_ISL_1139076_A/common_kestrel/Netherlands/20017381−001/2020_H5N8_2020−11−05
EPI_ISL_1139044_A/eurasian_oystercatcher/Netherlands/20017557−003/2020_H5N8_2020−11−10
EPI_ISL_1139108_A/mute_swan/Netherlands/20018754−004/2020_H5N8_2020−11−24
EPI_ISL_1139169_A/peregrine_falcon/Netherlands/20018821−002/2020_H5N8_2020−11−25
EPI_ISL_1139159_A/greylag_goose/Netherlands/20017058−002/2020_H5N8_2020−10−25
EPI_ISL_1139084_A/eurasian_wigeon/Netherlands/20016896−025/2020_H5N8_2020−11−02
EPI_ISL_775251_A/peregrine_falcon/Netherlands/20020038−001/2020_H5N8_2020−12−13
EPI_ISL_1139162_A/black_swan/Netherlands/20018185−001/2020_H5N8_2020−11−16
EPI_ISL_1139097_A/wild_goose/Netherlands/20017761−002/2020_H5N8_2020−11−04
EPI_ISL_1575130_A/Mute_Swan/Netherlands/1/2020_H5N8_2020−11−06
EPI_ISL_7267247_A/mute_swan/Netherlands/21039202−001/2021_H5N1_2021−11−20
EPI_ISL_10993074_A/Mallard/Netherlands/2/2022_H5N1_2022−02−14
EPI_ISL_5942524_A/chicken/Netherlands/21037750−001005/2021_H5N1_2021−10−31
EPI_ISL_6761006_A/mute_swan/Netherlands/21038362−001/2021_H5N1_2021−11−09
EPI_ISL_8799314_A/red_knot/Netherlands/21040956−003/2021_H5N1_2021−12−17
EPI_ISL_6761011_A/peregrine_falcon/Netherlands/21038169−002/2021_H5N1_2021−11−04
EPI_ISL_7996369_A/mute_swan/Netherlands/21039291−002/2021_H5N1_2021−11−19
EPI_ISL_9261740_A/Mallard/Netherlands/17/2021_H5N3_2021−12−29
EPI_ISL_7267254_A/greylag_goose/Netherlands/21039376−001/2021_H5N1_2021−11−23
EPI_ISL_6761018_A/mute_swan/Netherlands/21038537−004/2021_H5N1_2021−11−11
EPI_ISL_2174084_A/common_murre/Netherlands/21025491−002/2021_H5N1_2021−03−17
EPI_ISL_9616183_A/peregrine_falcon/Netherlands/22000191−001/2022_H5N1_2022−01−03
EPI_ISL_15909904_A/Barnacle_Goose/Netherlands/22019555−001/2022_H5N1_2022−10−31
EPI_ISL_6761005_A/chicken/Netherlands/21038211−001/2021_H5N1_2021−11−05
Root
98.1/99
87.1/95
100/100
0.007
Season 2020-2021
Season 2022-2023
Peregrine Falcon
Season 2021-2022
Season 2023-2024

PB1

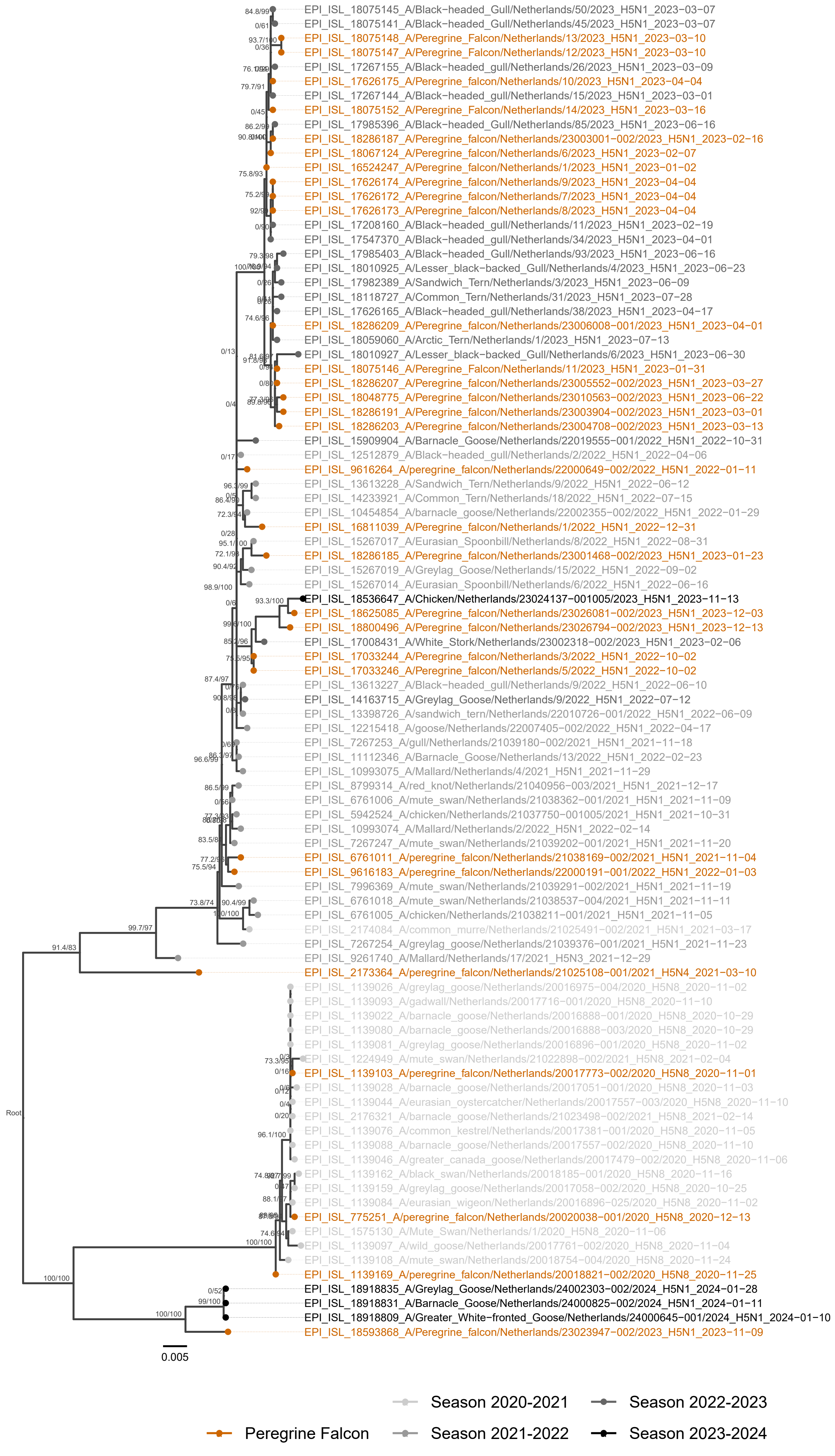
EPI_ISL_18075145_A/Black−headed_Gull/Netherlands/50/2023_H5N1_2023−03−07
EPI_ISL_18075141_A/Black−headed_Gull/Netherlands/45/2023_H5N1_2023−03−07
EPI_ISL_18075148_A/Peregrine_Falcon/Netherlands/13/2023_H5N1_2023−03−10
EPI_ISL_18075147_A/Peregrine_Falcon/Netherlands/12/2023_H5N1_2023−03−10
EPI_ISL_17267155_A/Black−headed_gull/Netherlands/26/2023_H5N1_2023−03−09
EPI_ISL_17626175_A/Peregrine_falcon/Netherlands/10/2023_H5N1_2023−04−04
EPI_ISL_17267144_A/Black−headed_gull/Netherlands/15/2023_H5N1_2023−03−01
EPI_ISL_18075152_A/Peregrine_Falcon/Netherlands/14/2023_H5N1_2023−03−16
EPI_ISL_17985396_A/Black−headed_Gull/Netherlands/85/2023_H5N1_2023−06−16
EPI_ISL_18286187_A/Peregrine_falcon/Netherlands/23003001−002/2023_H5N1_2023−02−16
EPI_ISL_18067124_A/Peregrine_falcon/Netherlands/6/2023_H5N1_2023−02−07
EPI_ISL_16524247_A/Peregrine_falcon/Netherlands/1/2023_H5N1_2023−01−02
EPI_ISL_17626174_A/Peregrine_falcon/Netherlands/9/2023_H5N1_2023−04−04
EPI_ISL_17626172_A/Peregrine_falcon/Netherlands/7/2023_H5N1_2023−04−04
EPI_ISL_17626173_A/Peregrine_falcon/Netherlands/8/2023_H5N1_2023−04−04
EPI_ISL_17208160_A/Black−headed_gull/Netherlands/11/2023_H5N1_2023−02−19
EPI_ISL_17547370_A/Black−headed_gull/Netherlands/34/2023_H5N1_2023−04−01
EPI_ISL_17985403_A/Black−headed_Gull/Netherlands/93/2023_H5N1_2023−06−16
EPI_ISL_18010925_A/Lesser_black−backed_Gull/Netherlands/4/2023_H5N1_2023−06−23
EPI_ISL_17982389_A/Sandwich_Tern/Netherlands/3/2023_H5N1_2023−06−09
EPI_ISL_18118727_A/Common_Tern/Netherlands/31/2023_H5N1_2023−07−28
EPI_ISL_17626165_A/Black−headed_gull/Netherlands/38/2023_H5N1_2023−04−17
EPI_ISL_18286209_A/Peregrine_falcon/Netherlands/23006008−001/2023_H5N1_2023−04−01
EPI_ISL_18059060_A/Arctic_Tern/Netherlands/1/2023_H5N1_2023−07−13
EPI_ISL_18010927_A/Lesser_black−backed_Gull/Netherlands/6/2023_H5N1_2023−06−30
EPI_ISL_18075146_A/Peregrine_Falcon/Netherlands/11/2023_H5N1_2023−01−31
EPI_ISL_18286207_A/Peregrine_falcon/Netherlands/23005552−002/2023_H5N1_2023−03−27
EPI_ISL_18048775_A/Peregrine_falcon/Netherlands/23010563−002/2023_H5N1_2023−06−22
EPI_ISL_18286191_A/Peregrine_falcon/Netherlands/23003904−002/2023_H5N1_2023−03−01
EPI_ISL_18286203_A/Peregrine_falcon/Netherlands/23004708−002/2023_H5N1_2023−03−13
EPI_ISL_15909904_A/Barnacle_Goose/Netherlands/22019555−001/2022_H5N1_2022−10−31
EPI_ISL_12512879_A/Black−headed_gull/Netherlands/2/2022_H5N1_2022−04−06
EPI_ISL_9616264_A/peregrine_falcon/Netherlands/22000649−002/2022_H5N1_2022−01−11
EPI_ISL_13613228_A/Sandwich_Tern/Netherlands/9/2022_H5N1_2022−06−12
EPI_ISL_14233921_A/Common_Tern/Netherlands/18/2022_H5N1_2022−07−15
EPI_ISL_10454854_A/barnacle_goose/Netherlands/22002355−002/2022_H5N1_2022−01−29
EPI_ISL_16811039_A/Peregrine_falcon/Netherlands/1/2022_H5N1_2022−12−31
EPI_ISL_15267017_A/Eurasian_Spoonbill/Netherlands/8/2022_H5N1_2022−08−31
EPI_ISL_18286185_A/Peregrine_falcon/Netherlands/23001468−002/2023_H5N1_2023−01−23
EPI_ISL_15267019_A/Greylag_Goose/Netherlands/15/2022_H5N1_2022−09−02
EPI_ISL_15267014_A/Eurasian_Spoonbill/Netherlands/6/2022_H5N1_2022−06−16
EPI_ISL_18536647_A/Chicken/Netherlands/23024137−001005/2023_H5N1_2023−11−13
EPI_ISL_18625085_A/Peregrine_falcon/Netherlands/23026081−002/2023_H5N1_2023−12−03
EPI_ISL_18800496_A/Peregrine_falcon/Netherlands/23026794−002/2023_H5N1_2023−12−13
EPI_ISL_17008431_A/White_Stork/Netherlands/23002318−002/2023_H5N1_2023−02−06
EPI_ISL_17033244_A/Peregrine_falcon/Netherlands/3/2022_H5N1_2022−10−02
EPI_ISL_17033246_A/Peregrine_falcon/Netherlands/5/2022_H5N1_2022−10−02
EPI_ISL_13613227_A/Black−headed_gull/Netherlands/9/2022_H5N1_2022−06−10
EPI_ISL_14163715_A/Greylag_Goose/Netherlands/9/2022_H5N1_2022−07−12
EPI_ISL_13398726_A/sandwich_tern/Netherlands/22010726−001/2022_H5N1_2022−06−09
EPI_ISL_12215418_A/goose/Netherlands/22007405−002/2022_H5N1_2022−04−17
EPI_ISL_7267253_A/gull/Netherlands/21039180−002/2021_H5N1_2021−11−18
EPI_ISL_11112346_A/Barnacle_Goose/Netherlands/13/2022_H5N1_2022−02−23
EPI_ISL_10993075_A/Mallard/Netherlands/4/2021_H5N1_2021−11−29
EPI_ISL_8799314_A/red_knot/Netherlands/21040956−003/2021_H5N1_2021−12−17
EPI_ISL_6761006_A/mute_swan/Netherlands/21038362−001/2021_H5N1_2021−11−09
EPI_ISL_5942524_A/chicken/Netherlands/21037750−001005/2021_H5N1_2021−10−31
EPI_ISL_10993074_A/Mallard/Netherlands/2/2022_H5N1_2022−02−14
EPI_ISL_7267247_A/mute_swan/Netherlands/21039202−001/2021_H5N1_2021−11−20
EPI_ISL_6761011_A/peregrine_falcon/Netherlands/21038169−002/2021_H5N1_2021−11−04
EPI_ISL_9616183_A/peregrine_falcon/Netherlands/22000191−001/2022_H5N1_2022−01−03
EPI_ISL_7996369_A/mute_swan/Netherlands/21039291−002/2021_H5N1_2021−11−19
EPI_ISL_6761018_A/mute_swan/Netherlands/21038537−004/2021_H5N1_2021−11−11
EPI_ISL_6761005_A/chicken/Netherlands/21038211−001/2021_H5N1_2021−11−05
EPI_ISL_2174084_A/common_murre/Netherlands/21025491−002/2021_H5N1_2021−03−17
EPI_ISL_7267254_A/greylag_goose/Netherlands/21039376−001/2021_H5N1_2021−11−23
EPI_ISL_9261740_A/Mallard/Netherlands/17/2021_H5N3_2021−12−29
EPI_ISL_2173364_A/peregrine_falcon/Netherlands/21025108−001/2021_H5N4_2021−03−10
EPI_ISL_1139026_A/greylag_goose/Netherlands/20016975−004/2020_H5N8_2020−11−02
EPI_ISL_1139093_A/gadwall/Netherlands/20017716−001/2020_H5N8_2020−11−10
EPI_ISL_1139022_A/barnacle_goose/Netherlands/20016888−001/2020_H5N8_2020−10−29
EPI_ISL_1139080_A/barnacle_goose/Netherlands/20016888−003/2020_H5N8_2020−10−29
EPI_ISL_1139081_A/greylag_goose/Netherlands/20016896−001/2020_H5N8_2020−11−02
EPI_ISL_1224949_A/mute_swan/Netherlands/21022898−002/2021_H5N8_2021−02−04
EPI_ISL_1139103_A/peregrine_falcon/Netherlands/20017773−002/2020_H5N8_2020−11−01
EPI_ISL_1139028_A/barnacle_goose/Netherlands/20017051−001/2020_H5N8_2020−11−03
EPI_ISL_1139044_A/eurasian_oystercatcher/Netherlands/20017557−003/2020_H5N8_2020−11−10
EPI_ISL_2176321_A/barnacle_goose/Netherlands/21023498−002/2021_H5N8_2021−02−14
EPI_ISL_1139076_A/common_kestrel/Netherlands/20017381−001/2020_H5N8_2020−11−05
EPI_ISL_1139088_A/barnacle_goose/Netherlands/20017557−002/2020_H5N8_2020−11−10
EPI_ISL_1139046_A/greater_canada_goose/Netherlands/20017479−002/2020_H5N8_2020−11−06
EPI_ISL_1139162_A/black_swan/Netherlands/20018185−001/2020_H5N8_2020−11−16
EPI_ISL_1139159_A/greylag_goose/Netherlands/20017058−002/2020_H5N8_2020−10−25
EPI_ISL_1139084_A/eurasian_wigeon/Netherlands/20016896−025/2020_H5N8_2020−11−02
EPI_ISL_775251_A/peregrine_falcon/Netherlands/20020038−001/2020_H5N8_2020−12−13
EPI_ISL_1575130_A/Mute_Swan/Netherlands/1/2020_H5N8_2020−11−06
EPI_ISL_1139097_A/wild_goose/Netherlands/20017761−002/2020_H5N8_2020−11−04
EPI_ISL_1139108_A/mute_swan/Netherlands/20018754−004/2020_H5N8_2020−11−24
EPI_ISL_1139169_A/peregrine_falcon/Netherlands/20018821−002/2020_H5N8_2020−11−25
EPI_ISL_18918835_A/Greylag_Goose/Netherlands/24002303−002/2024_H5N1_2024−01−28
EPI_ISL_18918831_A/Barnacle_Goose/Netherlands/24000825−002/2024_H5N1_2024−01−11
EPI_ISL_18918809_A/Greater_White−fronted_Goose/Netherlands/24000645−001/2024_H5N1_2024−01−10
EPI_ISL_18593868_A/Peregrine_falcon/Netherlands/23023947−002/2023_H5N1_2023−11−09
Root
0.005
Season 2020-2021
Season 2022-2023
Peregrine Falcon
Season 2021-2022
Season 2023-2024

PA

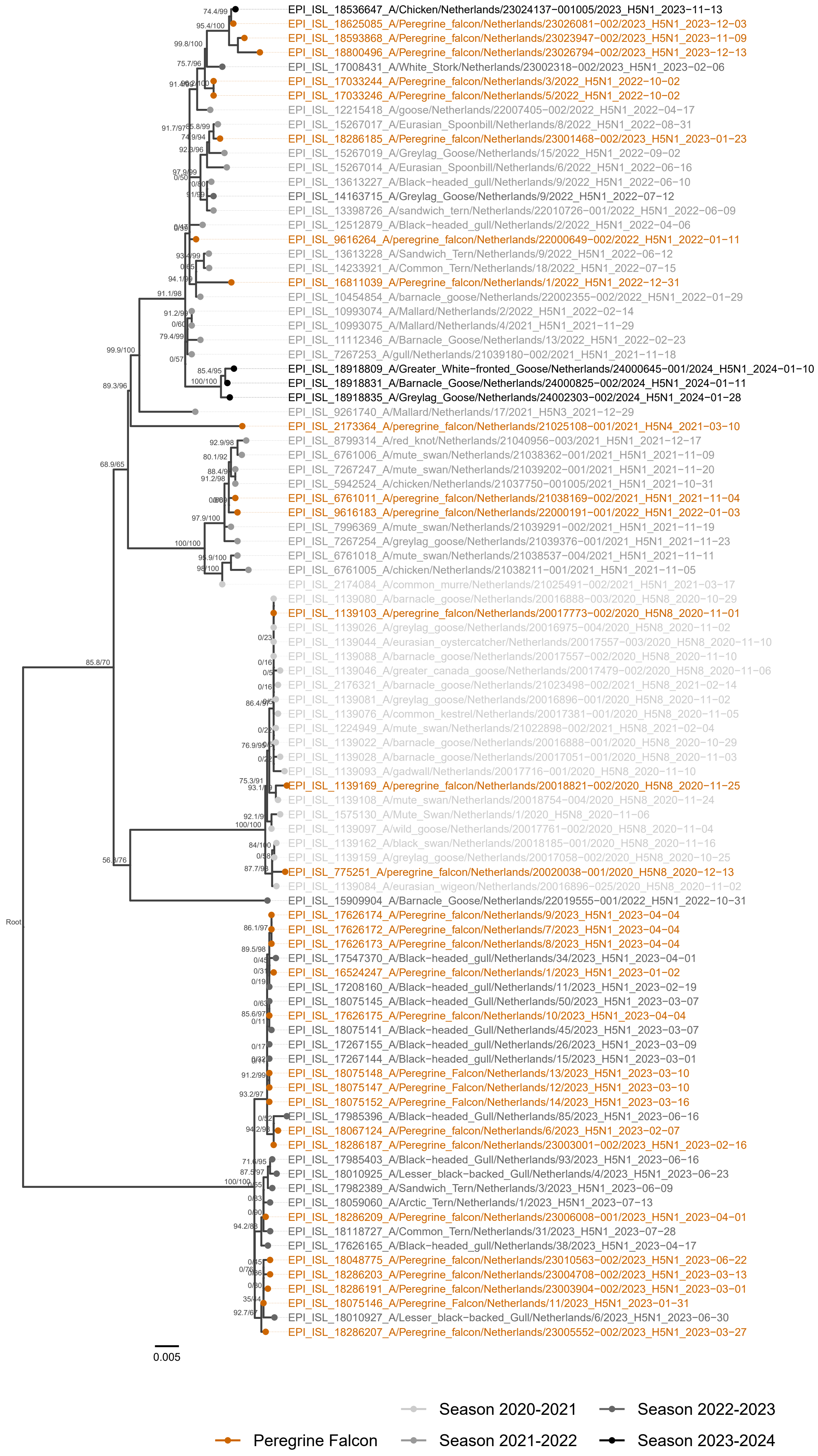
EPI_ISL_18536647_A/Chicken/Netherlands/23024137−001005/2023_H5N1_2023−11−13
EPI_ISL_18625085_A/Peregrine_falcon/Netherlands/23026081−002/2023_H5N1_2023−12−03
EPI_ISL_18593868_A/Peregrine_falcon/Netherlands/23023947−002/2023_H5N1_2023−11−09
EPI_ISL_18800496_A/Peregrine_falcon/Netherlands/23026794−002/2023_H5N1_2023−12−13
EPI_ISL_17008431_A/White_Stork/Netherlands/23002318−002/2023_H5N1_2023−02−06
EPI_ISL_17033244_A/Peregrine_falcon/Netherlands/3/2022_H5N1_2022−10−02
EPI_ISL_17033246_A/Peregrine_falcon/Netherlands/5/2022_H5N1_2022−10−02
EPI_ISL_12215418_A/goose/Netherlands/22007405−002/2022_H5N1_2022−04−17
EPI_ISL_15267017_A/Eurasian_Spoonbill/Netherlands/8/2022_H5N1_2022−08−31
EPI_ISL_18286185_A/Peregrine_falcon/Netherlands/23001468−002/2023_H5N1_2023−01−23
EPI_ISL_15267019_A/Greylag_Goose/Netherlands/15/2022_H5N1_2022−09−02
EPI_ISL_15267014_A/Eurasian_Spoonbill/Netherlands/6/2022_H5N1_2022−06−16
EPI_ISL_13613227_A/Black−headed_gull/Netherlands/9/2022_H5N1_2022−06−10
EPI_ISL_14163715_A/Greylag_Goose/Netherlands/9/2022_H5N1_2022−07−12
EPI_ISL_13398726_A/sandwich_tern/Netherlands/22010726−001/2022_H5N1_2022−06−09
EPI_ISL_12512879_A/Black−headed_gull/Netherlands/2/2022_H5N1_2022−04−06
EPI_ISL_9616264_A/peregrine_falcon/Netherlands/22000649−002/2022_H5N1_2022−01−11
EPI_ISL_13613228_A/Sandwich_Tern/Netherlands/9/2022_H5N1_2022−06−12
EPI_ISL_14233921_A/Common_Tern/Netherlands/18/2022_H5N1_2022−07−15
EPI_ISL_16811039_A/Peregrine_falcon/Netherlands/1/2022_H5N1_2022−12−31
EPI_ISL_10454854_A/barnacle_goose/Netherlands/22002355−002/2022_H5N1_2022−01−29
EPI_ISL_10993074_A/Mallard/Netherlands/2/2022_H5N1_2022−02−14
EPI_ISL_10993075_A/Mallard/Netherlands/4/2021_H5N1_2021−11−29
EPI_ISL_11112346_A/Barnacle_Goose/Netherlands/13/2022_H5N1_2022−02−23
EPI_ISL_7267253_A/gull/Netherlands/21039180−002/2021_H5N1_2021−11−18
EPI_ISL_18918809_A/Greater_White−fronted_Goose/Netherlands/24000645−001/2024_H5N1_2024−01−10
EPI_ISL_18918831_A/Barnacle_Goose/Netherlands/24000825−002/2024_H5N1_2024−01−11
EPI_ISL_18918835_A/Greylag_Goose/Netherlands/24002303−002/2024_H5N1_2024−01−28
EPI_ISL_9261740_A/Mallard/Netherlands/17/2021_H5N3_2021−12−29
EPI_ISL_2173364_A/peregrine_falcon/Netherlands/21025108−001/2021_H5N4_2021−03−10
EPI_ISL_8799314_A/red_knot/Netherlands/21040956−003/2021_H5N1_2021−12−17
EPI_ISL_6761006_A/mute_swan/Netherlands/21038362−001/2021_H5N1_2021−11−09
EPI_ISL_7267247_A/mute_swan/Netherlands/21039202−001/2021_H5N1_2021−11−20
EPI_ISL_5942524_A/chicken/Netherlands/21037750−001005/2021_H5N1_2021−10−31
EPI_ISL_6761011_A/peregrine_falcon/Netherlands/21038169−002/2021_H5N1_2021−11−04
EPI_ISL_9616183_A/peregrine_falcon/Netherlands/22000191−001/2022_H5N1_2022−01−03
EPI_ISL_7996369_A/mute_swan/Netherlands/21039291−002/2021_H5N1_2021−11−19
EPI_ISL_7267254_A/greylag_goose/Netherlands/21039376−001/2021_H5N1_2021−11−23
EPI_ISL_6761018_A/mute_swan/Netherlands/21038537−004/2021_H5N1_2021−11−11
EPI_ISL_6761005_A/chicken/Netherlands/21038211−001/2021_H5N1_2021−11−05
EPI_ISL_2174084_A/common_murre/Netherlands/21025491−002/2021_H5N1_2021−03−17
EPI_ISL_1139080_A/barnacle_goose/Netherlands/20016888−003/2020_H5N8_2020−10−29
EPI_ISL_1139103_A/peregrine_falcon/Netherlands/20017773−002/2020_H5N8_2020−11−01
EPI_ISL_1139026_A/greylag_goose/Netherlands/20016975−004/2020_H5N8_2020−11−02
EPI_ISL_1139044_A/eurasian_oystercatcher/Netherlands/20017557−003/2020_H5N8_2020−11−10
EPI_ISL_1139088_A/barnacle_goose/Netherlands/20017557−002/2020_H5N8_2020−11−10
EPI_ISL_1139046_A/greater_canada_goose/Netherlands/20017479−002/2020_H5N8_2020−11−06
EPI_ISL_2176321_A/barnacle_goose/Netherlands/21023498−002/2021_H5N8_2021−02−14
EPI_ISL_1139081_A/greylag_goose/Netherlands/20016896−001/2020_H5N8_2020−11−02
EPI_ISL_1139076_A/common_kestrel/Netherlands/20017381−001/2020_H5N8_2020−11−05
EPI_ISL_1224949_A/mute_swan/Netherlands/21022898−002/2021_H5N8_2021−02−04
EPI_ISL_1139022_A/barnacle_goose/Netherlands/20016888−001/2020_H5N8_2020−10−29
EPI_ISL_1139028_A/barnacle_goose/Netherlands/20017051−001/2020_H5N8_2020−11−03
EPI_ISL_1139093_A/gadwall/Netherlands/20017716−001/2020_H5N8_2020−11−10
EPI_ISL_1139169_A/peregrine_falcon/Netherlands/20018821−002/2020_H5N8_2020−11−25
EPI_ISL_1139108_A/mute_swan/Netherlands/20018754−004/2020_H5N8_2020−11−24
EPI_ISL_1575130_A/Mute_Swan/Netherlands/1/2020_H5N8_2020−11−06
EPI_ISL_1139097_A/wild_goose/Netherlands/20017761−002/2020_H5N8_2020−11−04
EPI_ISL_1139162_A/black_swan/Netherlands/20018185−001/2020_H5N8_2020−11−16
EPI_ISL_1139159_A/greylag_goose/Netherlands/20017058−002/2020_H5N8_2020−10−25
EPI_ISL_775251_A/peregrine_falcon/Netherlands/20020038−001/2020_H5N8_2020−12−13
EPI_ISL_1139084_A/eurasian_wigeon/Netherlands/20016896−025/2020_H5N8_2020−11−02
EPI_ISL_15909904_A/Barnacle_Goose/Netherlands/22019555−001/2022_H5N1_2022−10−31
EPI_ISL_17626174_A/Peregrine_falcon/Netherlands/9/2023_H5N1_2023−04−04
EPI_ISL_17626172_A/Peregrine_falcon/Netherlands/7/2023_H5N1_2023−04−04
EPI_ISL_17626173_A/Peregrine_falcon/Netherlands/8/2023_H5N1_2023−04−04
EPI_ISL_17547370_A/Black−headed_gull/Netherlands/34/2023_H5N1_2023−04−01
EPI_ISL_16524247_A/Peregrine_falcon/Netherlands/1/2023_H5N1_2023−01−02
EPI_ISL_17208160_A/Black−headed_gull/Netherlands/11/2023_H5N1_2023−02−19
EPI_ISL_18075145_A/Black−headed_Gull/Netherlands/50/2023_H5N1_2023−03−07
EPI_ISL_17626175_A/Peregrine_falcon/Netherlands/10/2023_H5N1_2023−04−04
EPI_ISL_18075141_A/Black−headed_Gull/Netherlands/45/2023_H5N1_2023−03−07
EPI_ISL_17267155_A/Black−headed_gull/Netherlands/26/2023_H5N1_2023−03−09
EPI_ISL_17267144_A/Black−headed_gull/Netherlands/15/2023_H5N1_2023−03−01
EPI_ISL_18075148_A/Peregrine_Falcon/Netherlands/13/2023_H5N1_2023−03−10
EPI_ISL_18075147_A/Peregrine_Falcon/Netherlands/12/2023_H5N1_2023−03−10
EPI_ISL_18075152_A/Peregrine_Falcon/Netherlands/14/2023_H5N1_2023−03−16
EPI_ISL_17985396_A/Black−headed_Gull/Netherlands/85/2023_H5N1_2023−06−16
EPI_ISL_18067124_A/Peregrine_falcon/Netherlands/6/2023_H5N1_2023−02−07
EPI_ISL_18286187_A/Peregrine_falcon/Netherlands/23003001−002/2023_H5N1_2023−02−16
EPI_ISL_17985403_A/Black−headed_Gull/Netherlands/93/2023_H5N1_2023−06−16
EPI_ISL_18010925_A/Lesser_black−backed_Gull/Netherlands/4/2023_H5N1_2023−06−23
EPI_ISL_17982389_A/Sandwich_Tern/Netherlands/3/2023_H5N1_2023−06−09
EPI_ISL_18059060_A/Arctic_Tern/Netherlands/1/2023_H5N1_2023−07−13
EPI_ISL_18286209_A/Peregrine_falcon/Netherlands/23006008−001/2023_H5N1_2023−04−01
EPI_ISL_18118727_A/Common_Tern/Netherlands/31/2023_H5N1_2023−07−28
EPI_ISL_17626165_A/Black−headed_gull/Netherlands/38/2023_H5N1_2023−04−17
EPI_ISL_18048775_A/Peregrine_falcon/Netherlands/23010563−002/2023_H5N1_2023−06−22
EPI_ISL_18286203_A/Peregrine_falcon/Netherlands/23004708−002/2023_H5N1_2023−03−13
EPI_ISL_18286191_A/Peregrine_falcon/Netherlands/23003904−002/2023_H5N1_2023−03−01
EPI_ISL_18075146_A/Peregrine_Falcon/Netherlands/11/2023_H5N1_2023−01−31
EPI_ISL_18010927_A/Lesser_black−backed_Gull/Netherlands/6/2023_H5N1_2023−06−30
EPI_ISL_18286207_A/Peregrine_falcon/Netherlands/23005552−002/2023_H5N1_2023−03−27
Root
0.005
Season 2020-2021
Season 2022-2023
Peregrine Falcon
Season 2021-2022
Season 2023-2024

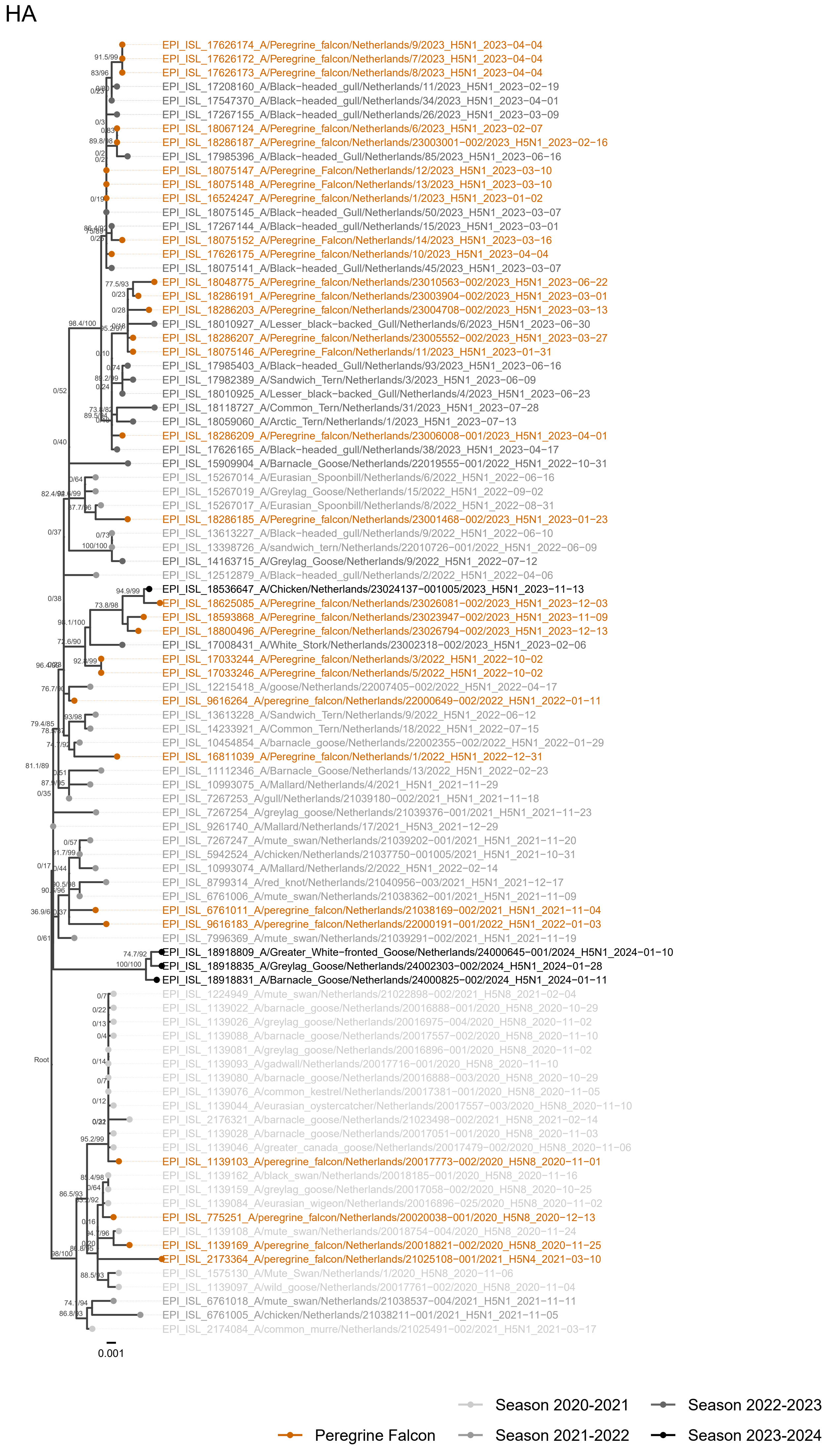

HA
EPI_ISL_17626174_A/Peregrine_falcon/Netherlands/9/2023_H5N1_2023-04-04
EPI_ISL_17626172_A/Peregrine_falcon/Netherlands/7/2023_H5N1_2023-04-04
EPI_ISL_17626173_A/Peregrine_falcon/Netherlands/8/2023_H5N1_2023-04-04
EPI_ISL_17208160_A/Black-headed_gull/Netherlands/11/2023_H5N1_2023-02-19
EPI_ISL_17547370_A/Black-headed_gull/Netherlands/34/2023_H5N1_2023-04-01
EPI_ISL_17267155_A/Black-headed_gull/Netherlands/26/2023_H5N1_2023-03-09
EPI_ISL_18067124_A/Peregrine_falcon/Netherlands/6/2023_H5N1_2023-02-07
EPI_ISL_18286187_A/Peregrine_falcon/Netherlands/23003001-002/2023_H5N1_2023-02-16
EPI_ISL_17985396_A/Black-headed_Gull/Netherlands/85/2023_H5N1_2023-06-16
EPI_ISL_18075147_A/Peregrine_Falcon/Netherlands/12/2023_H5N1_2023-03-10
EPI_ISL_18075148_A/Peregrine_Falcon/Netherlands/13/2023_H5N1_2023-03-10
EPI_ISL_16524247_A/Peregrine_falcon/Netherlands/1/2023_H5N1_2023-01-02
EPI_ISL_18075145_A/Black-headed_Gull/Netherlands/50/2023_H5N1_2023-03-07
EPI_ISL_17267144_A/Black-headed_gull/Netherlands/15/2023_H5N1_2023-03-01
EPI_ISL_18075152_A/Peregrine_Falcon/Netherlands/14/2023_H5N1_2023-03-16
EPI_ISL_17626175_A/Peregrine_falcon/Netherlands/10/2023_H5N1_2023-04-04
EPI_ISL_18075141_A/Black-headed_Gull/Netherlands/45/2023_H5N1_2023-03-07
EPI_ISL_18048775_A/Peregrine_falcon/Netherlands/23010563-002/2023_H5N1_2023-06-22
EPI_ISL_18286191_A/Peregrine_falcon/Netherlands/23003904-002/2023_H5N1_2023-03-01
EPI_ISL_18286203_A/Peregrine_falcon/Netherlands/23004708-002/2023_H5N1_2023-03-13
EPI_ISL_18010927_A/Lesser_black-backed_Gull/Netherlands/6/2023_H5N1_2023-06-30
EPI_ISL_18286207_A/Peregrine_falcon/Netherlands/23005552-002/2023_H5N1_2023-03-27
EPI_ISL_18075146_A/Peregrine_Falcon/Netherlands/11/2023_H5N1_2023-01-31
EPI_ISL_17985403_A/Black-headed_Gull/Netherlands/93/2023_H5N1_2023-06-16
EPI_ISL_17982389_A/Sandwich_Tern/Netherlands/3/2023_H5N1_2023-06-09
EPI_ISL_18010925_A/Lesser_black-backed_Gull/Netherlands/4/2023_H5N1_2023-06-23
EPI_ISL_18118727_A/Common_Tern/Netherlands/31/2023_H5N1_2023-07-28
EPI_ISL_18059060_A/Arctic_Tern/Netherlands/1/2023_H5N1_2023-07-13
EPI_ISL_18286209_A/Peregrine_falcon/Netherlands/23006008-001/2023_H5N1_2023-04-01
EPI_ISL_17626165_A/Black-headed_gull/Netherlands/38/2023_H5N1_2023-04-17
EPI_ISL_15909904_A/Barnacle_Goose/Netherlands/22019555-001/2022_H5N1_2022-10-31
EPI_ISL_15267014_A/Eurasian_Spoonbill/Netherlands/6/2022_H5N1_2022-06-16
EPI_ISL_15267019_A/Greylag_Goose/Netherlands/15/2022_H5N1_2022-09-02
EPI_ISL_15267017_A/Eurasian_Spoonbill/Netherlands/8/2022_H5N1_2022-08-31
EPI_ISL_18286185_A/Peregrine_falcon/Netherlands/23001468-002/2023_H5N1_2023-01-23
EPI_ISL_13613227_A/Black-headed_gull/Netherlands/9/2022_H5N1_2022-06-10
EPI_ISL_13398726_A/sandwich_tern/Netherlands/22010726-001/2022_H5N1_2022-06-09
EPI_ISL_14163715_A/Greylag_Goose/Netherlands/9/2022_H5N1_2022-07-12
EPI_ISL_12512879_A/Black-headed_gull/Netherlands/2/2022_H5N1_2022-04-06
EPI_ISL_18536647_A/Chicken/Netherlands/23024137-001005/2023_H5N1_2023-11-13
EPI_ISL_18625085_A/Peregrine_falcon/Netherlands/23026081-002/2023_H5N1_2023-12-03
EPI_ISL_18593868_A/Peregrine_falcon/Netherlands/23023947-002/2023_H5N1_2023-11-09
EPI_ISL_18800496_A/Peregrine_falcon/Netherlands/23026794-002/2023_H5N1_2023-12-13
EPI_ISL_17008431_A/White_Stork/Netherlands/23002318-002/2023_H5N1_2023-02-06
EPI_ISL_17033244_A/Peregrine_falcon/Netherlands/3/2022_H5N1_2022-10-02
EPI_ISL_17033246_A/Peregrine_falcon/Netherlands/5/2022_H5N1_2022-10-02
EPI_ISL_12215418_A/goose/Netherlands/22007405-002/2022_H5N1_2022-04-17
EPI_ISL_9616264_A/peregrine_falcon/Netherlands/22000649-002/2022_H5N1_2022-01-11
EPI_ISL_13613228_A/Sandwich_Tern/Netherlands/9/2022_H5N1_2022-06-12
EPI_ISL_14233921_A/Common_Tern/Netherlands/18/2022_H5N1_2022-07-15
EPI_ISL_10454854_A/barnacle_goose/Netherlands/22002355-002/2022_H5N1_2022-01-29
EPI_ISL_16811039_A/Peregrine_falcon/Netherlands/1/2022_H5N1_2022-12-31
EPI_ISL_11112346_A/Barnacle_Goose/Netherlands/13/2022_H5N1_2022-02-23
EPI_ISL_10993075_A/Mallard/Netherlands/4/2021_H5N1_2021-11-29
EPI_ISL_7267253_A/gull/Netherlands/21039180-002/2021_H5N1_2021-11-18
EPI_ISL_7267254_A/greylag_goose/Netherlands/21039376-001/2021_H5N1_2021-11-23
EPI_ISL_9261740_A/Mallard/Netherlands/17/2021_H5N3_2021-12-29
EPI_ISL_7267247_A/mute_swan/Netherlands/21039202-001/2021_H5N1_2021-11-20
EPI_ISL_5942524_A/chicken/Netherlands/21037750-001005/2021_H5N1_2021-10-31
EPI_ISL_10993074_A/Mallard/Netherlands/2/2022_H5N1_2022-02-14
EPI_ISL_8799314_A/red_knot/Netherlands/21040956-003/2021_H5N1_2021-12-17
EPI_ISL_6761006_A/mute_swan/Netherlands/21038362-001/2021_H5N1_2021-11-09
EPI_ISL_6761011_A/peregrine_falcon/Netherlands/21038169-002/2021_H5N1_2021-11-04
EPI_ISL_9616183_A/peregrine_falcon/Netherlands/22000191-001/2022_H5N1_2022-01-03
EPI_ISL_7996369_A/mute_swan/Netherlands/21039291-002/2021_H5N1_2021-11-19
EPI_ISL_18918809_A/Greater_White-fronted_Goose/Netherlands/24000645-001/2024_H5N1_2024-01-10
EPI_ISL_18918835_A/Greylag_Goose/Netherlands/24002303-002/2024_H5N1_2024-01-28
EPI_ISL_18918831_A/Barnacle_Goose/Netherlands/24000825-002/2024_H5N1_2024-01-11
EPI_ISL_1224949_A/mute_swan/Netherlands/21022898-002/2021_H5N8_2021-02-04
EPI_ISL_1139022_A/barnacle_goose/Netherlands/20016888-001/2020_H5N8_2020-10-29
EPI_ISL_1139026_A/greylag_goose/Netherlands/20016975-004/2020_H5N8_2020-11-02
EPI_ISL_1139088_A/barnacle_goose/Netherlands/20017557-002/2020_H5N8_2020-11-10
EPI_ISL_1139081_A/greylag_goose/Netherlands/20016896-001/2020_H5N8_2020-11-02
EPI_ISL_1139093_A/gadwall/Netherlands/20017716-001/2020_H5N8_2020-11-10
EPI_ISL_1139080_A/barnacle_goose/Netherlands/20016888-003/2020_H5N8_2020-10-29
EPI_ISL_1139076_A/common_kestrel/Netherlands/20017381-001/2020_H5N8_2020-11-05
EPI_ISL_1139044_A/eurasian_oystercatcher/Netherlands/20017557-003/2020_H5N8_2020-11-10
EPI_ISL_2176321_A/barnacle_goose/Netherlands/21023498-002/2021_H5N8_2021-02-14
EPI_ISL_1139028_A/barnacle_goose/Netherlands/20017051-001/2020_H5N8_2020-11-03
EPI_ISL_1139046_A/greater_canada_goose/Netherlands/20017479-002/2020_H5N8_2020-11-06
EPI_ISL_1139103_A/peregrine_falcon/Netherlands/20017773-002/2020_H5N8_2020-11-01
EPI_ISL_1139162_A/black_swan/Netherlands/20018185-001/2020_H5N8_2020-11-16
EPI_ISL_1139159_A/greylag_goose/Netherlands/20017058-002/2020_H5N8_2020-10-25
EPI_ISL_1139084_A/eurasian_wigeon/Netherlands/20016896-025/2020_H5N8_2020-11-02
EPI_ISL_775251_A/peregrine_falcon/Netherlands/20020038-001/2020_H5N8_2020-12-13
EPI_ISL_1139108_A/mute_swan/Netherlands/20018754-004/2020_H5N8_2020-11-24
EPI_ISL_1139169_A/peregrine_falcon/Netherlands/20018821-002/2020_H5N8_2020-11-25
EPI_ISL_2173364_A/peregrine_falcon/Netherlands/21025108-001/2021_H5N4_2021-03-10
EPI_ISL_1575130_A/Mute_Swan/Netherlands/1/2020_H5N8_2020-11-06
EPI_ISL_1139097_A/wild_goose/Netherlands/20017761-002/2020_H5N8_2020-11-04
EPI_ISL_6761018_A/mute_swan/Netherlands/21038537-004/2021_H5N1_2021-11-11
EPI_ISL_6761005_A/chicken/Netherlands/21038211-001/2021_H5N1_2021-11-05
EPI_ISL_2174084_A/common_murre/Netherlands/21025491-002/2021_H5N1_2021-03-17
Root
0.001
Season 2020-2021
Season 2022-2023
Peregrine Falcon
Season 2021-2022
Season 2023-2024

NP

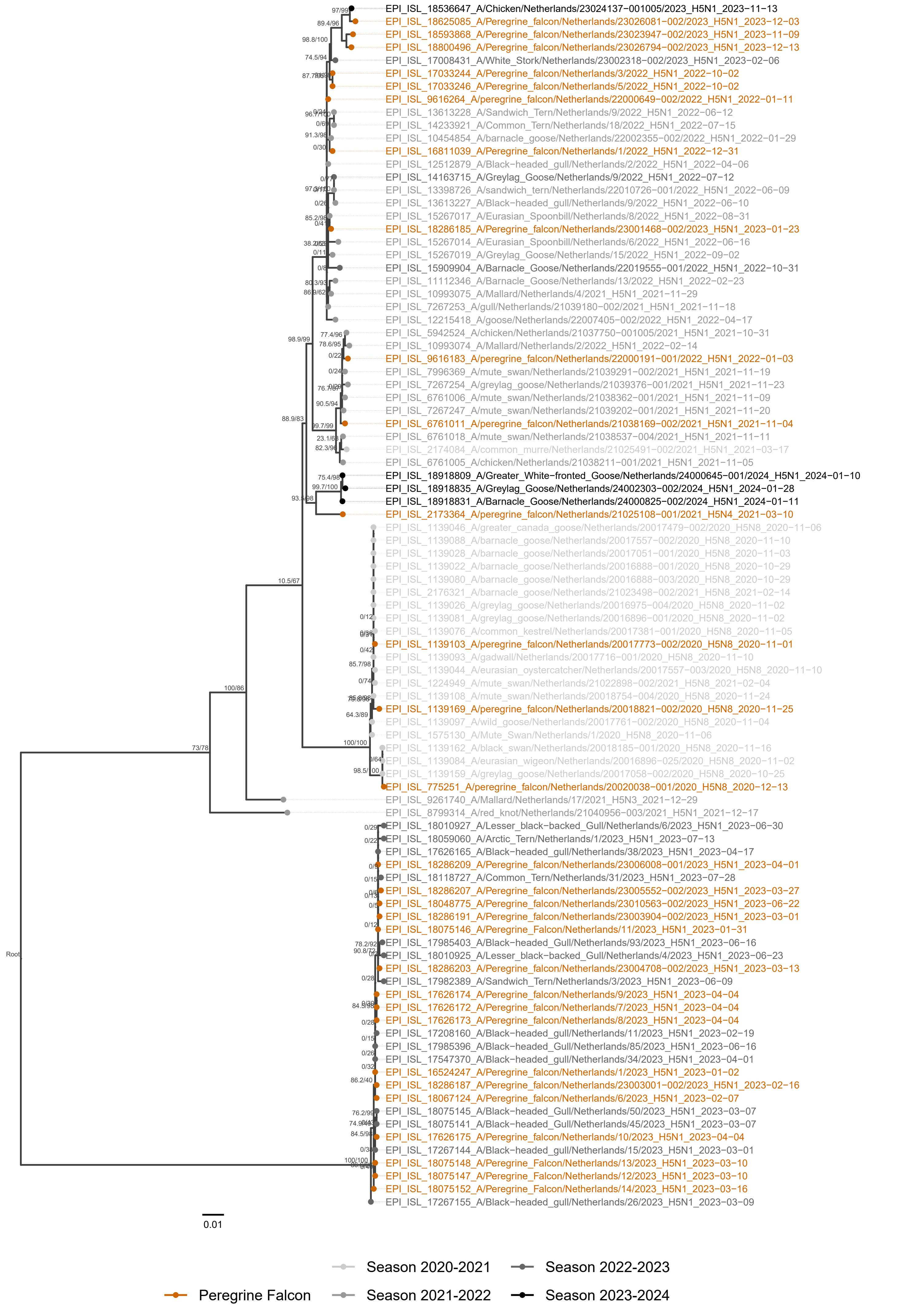
EPI_ISL_18536647_A/Chicken/Netherlands/23024137-001005/2023_H5N1_2023-11-13
EPI_ISL_18625085_A/Peregrine_falcon/Netherlands/23026081-002/2023_H5N1_2023-12-03
EPI_ISL_18593868_A/Peregrine_falcon/Netherlands/23023947-002/2023_H5N1_2023-11-09
EPI_ISL_18800496_A/Peregrine_falcon/Netherlands/23026794-002/2023_H5N1_2023-12-13
EPI_ISL_17008431_A/White_Stork/Netherlands/23002318-002/2023_H5N1_2023-02-06
EPI_ISL_17033244_A/Peregrine_falcon/Netherlands/3/2022_H5N1_2022-10-02
EPI_ISL_17033246_A/Peregrine_falcon/Netherlands/5/2022_H5N1_2022-10-02
EPI_ISL_9616264_A/peregrine_falcon/Netherlands/22000649-002/2022_H5N1_2022-01-11
EPI_ISL_13613228_A/Sandwich_Tern/Netherlands/9/2022_H5N1_2022-06-12
EPI_ISL_14233921_A/Common_Tern/Netherlands/18/2022_H5N1_2022-07-15
EPI_ISL_10454854_A/barnacle_goose/Netherlands/22002355-002/2022_H5N1_2022-01-29
EPI_ISL_16811039_A/Peregrine_falcon/Netherlands/1/2022_H5N1_2022-12-31
EPI_ISL_12512879_A/Black-headed_gull/Netherlands/2/2022_H5N1_2022-04-06
EPI_ISL_14163715_A/Greylag_Goose/Netherlands/9/2022_H5N1_2022-07-12
EPI_ISL_13398726_A/sandwich_tern/Netherlands/22010726-001/2022_H5N1_2022-06-09
EPI_ISL_13613227_A/Black-headed_gull/Netherlands/9/2022_H5N1_2022-06-10
EPI_ISL_15267017_A/Eurasian_Spoonbill/Netherlands/8/2022_H5N1_2022-08-31
EPI_ISL_18286185_A/Peregrine_falcon/Netherlands/23001468-002/2023_H5N1_2023-01-23
EPI_ISL_15267014_A/Eurasian_Spoonbill/Netherlands/6/2022_H5N1_2022-06-16
EPI_ISL_15267019_A/Greylag_Goose/Netherlands/15/2022_H5N1_2022-09-02
EPI_ISL_15909904_A/Barnacle_Goose/Netherlands/22019555-001/2022_H5N1_2022-10-31
EPI_ISL_11112346_A/Barnacle_Goose/Netherlands/13/2022_H5N1_2022-02-23
EPI_ISL_10993075_A/Mallard/Netherlands/4/2021_H5N1_2021-11-29
EPI_ISL_7267253_A/gull/Netherlands/21039180-002/2021_H5N1_2021-11-18
EPI_ISL_12215418_A/goose/Netherlands/22007405-002/2022_H5N1_2022-04-17
EPI_ISL_5942524_A/chicken/Netherlands/21037750-001005/2021_H5N1_2021-10-31
EPI_ISL_10993074_A/Mallard/Netherlands/2/2022_H5N1_2022-02-14
EPI_ISL_9616183_A/peregrine_falcon/Netherlands/22000191-001/2022_H5N1_2022-01-03
EPI_ISL_7996369_A/mute_swan/Netherlands/21039291-002/2021_H5N1_2021-11-19
EPI_ISL_7267254_A/greylag_goose/Netherlands/21039376-001/2021_H5N1_2021-11-23
EPI_ISL_6761006_A/mute_swan/Netherlands/21038362-001/2021_H5N1_2021-11-09
EPI_ISL_7267247_A/mute_swan/Netherlands/21039202-001/2021_H5N1_2021-11-20
EPI_ISL_6761011_A/peregrine_falcon/Netherlands/21038169-002/2021_H5N1_2021-11-04
EPI_ISL_6761018_A/mute_swan/Netherlands/21038537-004/2021_H5N1_2021-11-11
EPI_ISL_2174084_A/common_murre/Netherlands/21025491-002/2021_H5N1_2021-03-17
EPI_ISL_6761005_A/chicken/Netherlands/21038211-001/2021_H5N1_2021-11-05
EPI_ISL_18918809_A/Greater_White-fronted_Goose/Netherlands/24000645-001/2024_H5N1_2024-01-10
EPI_ISL_18918835_A/Greylag_Goose/Netherlands/24002303-002/2024_H5N1_2024-01-28
EPI_ISL_18918831_A/Barnacle_Goose/Netherlands/24000825-002/2024_H5N1_2024-01-11
EPI_ISL_2173364_A/peregrine_falcon/Netherlands/21025108-001/2021_H5N4_2021-03-10
EPI_ISL_1139046_A/greater_canada_goose/Netherlands/20017479-002/2020_H5N8_2020-11-06
EPI_ISL_1139088_A/barnacle_goose/Netherlands/20017557-002/2020_H5N8_2020-11-10
EPI_ISL_1139028_A/barnacle_goose/Netherlands/20017051-001/2020_H5N8_2020-11-03
EPI_ISL_1139022_A/barnacle_goose/Netherlands/20016888-001/2020_H5N8_2020-10-29
EPI_ISL_1139080_A/barnacle_goose/Netherlands/20016888-003/2020_H5N8_2020-10-29
EPI_ISL_2176321_A/barnacle_goose/Netherlands/21023498-002/2021_H5N8_2021-02-14
EPI_ISL_1139026_A/greylag_goose/Netherlands/20016975-004/2020_H5N8_2020-11-02
EPI_ISL_1139081_A/greylag_goose/Netherlands/20016896-001/2020_H5N8_2020-11-02
EPI_ISL_1139076_A/common_kestrel/Netherlands/20017381-001/2020_H5N8_2020-11-05
EPI_ISL_1139103_A/peregrine_falcon/Netherlands/20017773-002/2020_H5N8_2020-11-01
EPI_ISL_1139093_A/gadwall/Netherlands/20017716-001/2020_H5N8_2020-11-10
EPI_ISL_1139044_A/eurasian_oystercatcher/Netherlands/20017557-003/2020_H5N8_2020-11-10
EPI_ISL_1224949_A/mute_swan/Netherlands/21022898-002/2021_H5N8_2021-02-04
EPI_ISL_1139108_A/mute_swan/Netherlands/20018754-004/2020_H5N8_2020-11-24
EPI_ISL_1139169_A/peregrine_falcon/Netherlands/20018821-002/2020_H5N8_2020-11-25
EPI_ISL_1139097_A/wild_goose/Netherlands/20017761-002/2020_H5N8_2020-11-04
EPI_ISL_1575130_A/Mute_Swan/Netherlands/1/2020_H5N8_2020-11-06
EPI_ISL_1139162_A/black_swan/Netherlands/20018185-001/2020_H5N8_2020-11-16
EPI_ISL_1139084_A/eurasian_wigeon/Netherlands/20016896-025/2020_H5N8_2020-11-02
EPI_ISL_1139159_A/greylag_goose/Netherlands/20017058-002/2020_H5N8_2020-10-25
EPI_ISL_775251_A/peregrine_falcon/Netherlands/20020038-001/2020_H5N8_2020-12-13
EPI_ISL_9261740_A/Mallard/Netherlands/17/2021_H5N3_2021-12-29
EPI_ISL_8799314_A/red_knot/Netherlands/21040956-003/2021_H5N1_2021-12-17
EPI_ISL_18010927_A/Lesser_black-backed_Gull/Netherlands/6/2023_H5N1_2023-06-30
EPI_ISL_18059060_A/Arctic_Tern/Netherlands/1/2023_H5N1_2023-07-13
EPI_ISL_17626165_A/Black-headed_gull/Netherlands/38/2023_H5N1_2023-04-17
EPI_ISL_18286209_A/Peregrine_falcon/Netherlands/23006008-001/2023_H5N1_2023-04-01
EPI_ISL_18118727_A/Common_Tern/Netherlands/31/2023_H5N1_2023-07-28
EPI_ISL_18286207_A/Peregrine_falcon/Netherlands/23005552-002/2023_H5N1_2023-03-27
EPI_ISL_18048775_A/Peregrine_falcon/Netherlands/23010563-002/2023_H5N1_2023-06-22
EPI_ISL_18286191_A/Peregrine_falcon/Netherlands/23003904-002/2023_H5N1_2023-03-01
EPI_ISL_18075146_A/Peregrine_Falcon/Netherlands/11/2023_H5N1_2023-01-31
EPI_ISL_17985403_A/Black-headed_Gull/Netherlands/93/2023_H5N1_2023-06-16
EPI_ISL_18010925_A/Lesser_black-backed_Gull/Netherlands/4/2023_H5N1_2023-06-23
EPI_ISL_18286203_A/Peregrine_falcon/Netherlands/23004708-002/2023_H5N1_2023-03-13
EPI_ISL_17982389_A/Sandwich_Tern/Netherlands/3/2023_H5N1_2023-06-09
EPI_ISL_17626174_A/Peregrine_falcon/Netherlands/9/2023_H5N1_2023-04-04
EPI_ISL_17626172_A/Peregrine_falcon/Netherlands/7/2023_H5N1_2023-04-04
EPI_ISL_17626173_A/Peregrine_falcon/Netherlands/8/2023_H5N1_2023-04-04
EPI_ISL_17208160_A/Black-headed_gull/Netherlands/11/2023_H5N1_2023-02-19
EPI_ISL_17985396_A/Black-headed_Gull/Netherlands/85/2023_H5N1_2023-06-16
EPI_ISL_17547370_A/Black-headed_gull/Netherlands/34/2023_H5N1_2023-04-01
EPI_ISL_16524247_A/Peregrine_falcon/Netherlands/1/2023_H5N1_2023-01-02
EPI_ISL_18286187_A/Peregrine_falcon/Netherlands/23003001-002/2023_H5N1_2023-02-16
EPI_ISL_18067124_A/Peregrine_falcon/Netherlands/6/2023_H5N1_2023-02-07
EPI_ISL_18075145_A/Black-headed_Gull/Netherlands/50/2023_H5N1_2023-03-07
EPI_ISL_18075141_A/Black-headed_Gull/Netherlands/45/2023_H5N1_2023-03-07
EPI_ISL_17626175_A/Peregrine_falcon/Netherlands/10/2023_H5N1_2023-04-04
EPI_ISL_17267144_A/Black-headed_gull/Netherlands/15/2023_H5N1_2023-03-01
EPI_ISL_18075148_A/Peregrine_Falcon/Netherlands/13/2023_H5N1_2023-03-10
EPI_ISL_18075147_A/Peregrine_Falcon/Netherlands/12/2023_H5N1_2023-03-10
EPI_ISL_18075152_A/Peregrine_Falcon/Netherlands/14/2023_H5N1_2023-03-16
EPI_ISL_17267155_A/Black-headed_gull/Netherlands/26/2023_H5N1_2023-03-09
Root
0.01
Season 2020-2021
Season 2022-2023
Peregrine Falcon
Season 2021-2022
Season 2023-2024

NA_N1

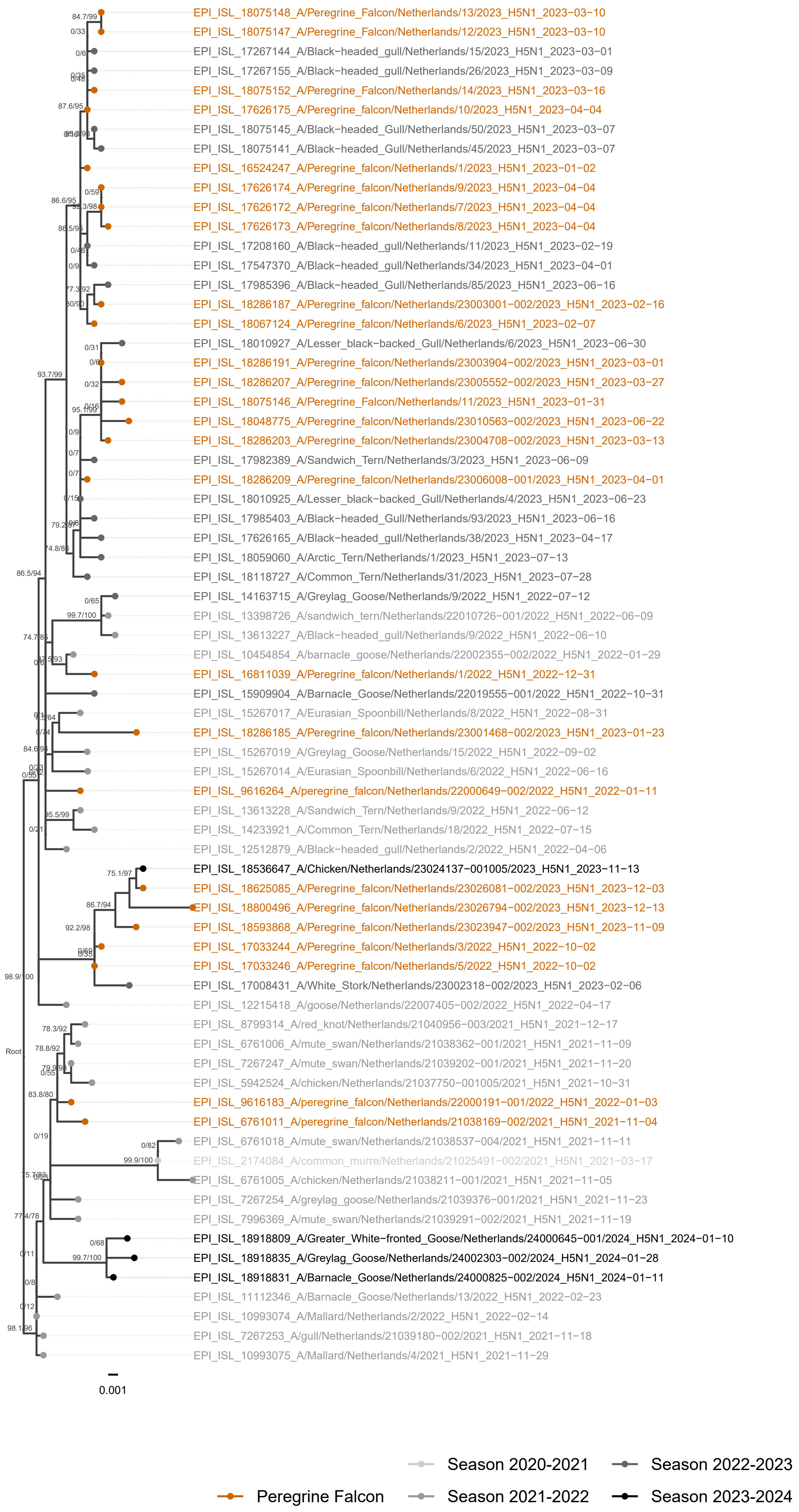
EPI_ISL_18075148_A/Peregrine_Falcon/Netherlands/13/2023_H5N1_2023−03−10
EPI_ISL_18075147_A/Peregrine_Falcon/Netherlands/12/2023_H5N1_2023−03−10
EPI_ISL_17267144_A/Black−headed_gull/Netherlands/15/2023_H5N1_2023−03−01
EPI_ISL_17267155_A/Black−headed_gull/Netherlands/26/2023_H5N1_2023−03−09
EPI_ISL_18075152_A/Peregrine_Falcon/Netherlands/14/2023_H5N1_2023−03−16
EPI_ISL_17626175_A/Peregrine_falcon/Netherlands/10/2023_H5N1_2023−04−04
EPI_ISL_18075145_A/Black−headed_Gull/Netherlands/50/2023_H5N1_2023−03−07
EPI_ISL_18075141_A/Black−headed_Gull/Netherlands/45/2023_H5N1_2023−03−07
EPI_ISL_16524247_A/Peregrine_falcon/Netherlands/1/2023_H5N1_2023−01−02
EPI_ISL_17626174_A/Peregrine_falcon/Netherlands/9/2023_H5N1_2023−04−04
EPI_ISL_17626172_A/Peregrine_falcon/Netherlands/7/2023_H5N1_2023−04−04
EPI_ISL_17626173_A/Peregrine_falcon/Netherlands/8/2023_H5N1_2023−04−04
EPI_ISL_17208160_A/Black−headed_gull/Netherlands/11/2023_H5N1_2023−02−19
EPI_ISL_17547370_A/Black−headed_gull/Netherlands/34/2023_H5N1_2023−04−01
EPI_ISL_17985396_A/Black−headed_Gull/Netherlands/85/2023_H5N1_2023−06−16
EPI_ISL_18286187_A/Peregrine_falcon/Netherlands/23003001−002/2023_H5N1_2023−02−16
EPI_ISL_18067124_A/Peregrine_falcon/Netherlands/6/2023_H5N1_2023−02−07
EPI_ISL_18010927_A/Lesser_black−backed_Gull/Netherlands/6/2023_H5N1_2023−06−30
EPI_ISL_18286191_A/Peregrine_falcon/Netherlands/23003904−002/2023_H5N1_2023−03−01
EPI_ISL_18286207_A/Peregrine_falcon/Netherlands/23005552−002/2023_H5N1_2023−03−27
EPI_ISL_18075146_A/Peregrine_Falcon/Netherlands/11/2023_H5N1_2023−01−31
EPI_ISL_18048775_A/Peregrine_falcon/Netherlands/23010563−002/2023_H5N1_2023−06−22
EPI_ISL_18286203_A/Peregrine_falcon/Netherlands/23004708−002/2023_H5N1_2023−03−13
EPI_ISL_17982389_A/Sandwich_Tern/Netherlands/3/2023_H5N1_2023−06−09
EPI_ISL_18286209_A/Peregrine_falcon/Netherlands/23006008−001/2023_H5N1_2023−04−01
EPI_ISL_18010925_A/Lesser_black−backed_Gull/Netherlands/4/2023_H5N1_2023−06−23
EPI_ISL_17985403_A/Black−headed_Gull/Netherlands/93/2023_H5N1_2023−06−16
EPI_ISL_17626165_A/Black−headed_gull/Netherlands/38/2023_H5N1_2023−04−17
EPI_ISL_18059060_A/Arctic_Tern/Netherlands/1/2023_H5N1_2023−07−13
EPI_ISL_18118727_A/Common_Tern/Netherlands/31/2023_H5N1_2023−07−28
EPI_ISL_14163715_A/Greylag_Goose/Netherlands/9/2022_H5N1_2022−07−12
EPI_ISL_13398726_A/sandwich_tern/Netherlands/22010726−001/2022_H5N1_2022−06−09
EPI_ISL_13613227_A/Black−headed_gull/Netherlands/9/2022_H5N1_2022−06−10
EPI_ISL_10454854_A/barnacle_goose/Netherlands/22002355−002/2022_H5N1_2022−01−29
EPI_ISL_16811039_A/Peregrine_falcon/Netherlands/1/2022_H5N1_2022−12−31
EPI_ISL_15909904_A/Barnacle_Goose/Netherlands/22019555−001/2022_H5N1_2022−10−31
EPI_ISL_15267017_A/Eurasian_Spoonbill/Netherlands/8/2022_H5N1_2022−08−31
EPI_ISL_18286185_A/Peregrine_falcon/Netherlands/23001468−002/2023_H5N1_2023−01−23
EPI_ISL_15267019_A/Greylag_Goose/Netherlands/15/2022_H5N1_2022−09−02
EPI_ISL_15267014_A/Eurasian_Spoonbill/Netherlands/6/2022_H5N1_2022−06−16
EPI_ISL_9616264_A/peregrine_falcon/Netherlands/22000649−002/2022_H5N1_2022−01−11
EPI_ISL_13613228_A/Sandwich_Tern/Netherlands/9/2022_H5N1_2022−06−12
EPI_ISL_14233921_A/Common_Tern/Netherlands/18/2022_H5N1_2022−07−15
EPI_ISL_12512879_A/Black−headed_gull/Netherlands/2/2022_H5N1_2022−04−06
EPI_ISL_18536647_A/Chicken/Netherlands/23024137−001005/2023_H5N1_2023−11−13
EPI_ISL_18625085_A/Peregrine_falcon/Netherlands/23026081−002/2023_H5N1_2023−12−03
EPI_ISL_18800496_A/Peregrine_falcon/Netherlands/23026794−002/2023_H5N1_2023−12−13
EPI_ISL_18593868_A/Peregrine_falcon/Netherlands/23023947−002/2023_H5N1_2023−11−09
EPI_ISL_17033244_A/Peregrine_falcon/Netherlands/3/2022_H5N1_2022−10−02
EPI_ISL_17033246_A/Peregrine_falcon/Netherlands/5/2022_H5N1_2022−10−02
EPI_ISL_17008431_A/White_Stork/Netherlands/23002318−002/2023_H5N1_2023−02−06
EPI_ISL_12215418_A/goose/Netherlands/22007405−002/2022_H5N1_2022−04−17
EPI_ISL_8799314_A/red_knot/Netherlands/21040956−003/2021_H5N1_2021−12−17
EPI_ISL_6761006_A/mute_swan/Netherlands/21038362−001/2021_H5N1_2021−11−09
EPI_ISL_7267247_A/mute_swan/Netherlands/21039202−001/2021_H5N1_2021−11−20
EPI_ISL_5942524_A/chicken/Netherlands/21037750−001005/2021_H5N1_2021−10−31
EPI_ISL_9616183_A/peregrine_falcon/Netherlands/22000191−001/2022_H5N1_2022−01−03
EPI_ISL_6761011_A/peregrine_falcon/Netherlands/21038169−002/2021_H5N1_2021−11−04
EPI_ISL_6761018_A/mute_swan/Netherlands/21038537−004/2021_H5N1_2021−11−11
EPI_ISL_2174084_A/common_murre/Netherlands/21025491−002/2021_H5N1_2021−03−17
EPI_ISL_6761005_A/chicken/Netherlands/21038211−001/2021_H5N1_2021−11−05
EPI_ISL_7267254_A/greylag_goose/Netherlands/21039376−001/2021_H5N1_2021−11−23
EPI_ISL_7996369_A/mute_swan/Netherlands/21039291−002/2021_H5N1_2021−11−19
EPI_ISL_18918809_A/Greater_White−fronted_Goose/Netherlands/24000645−001/2024_H5N1_2024−01−10
EPI_ISL_18918835_A/Greylag_Goose/Netherlands/24002303−002/2024_H5N1_2024−01−28
EPI_ISL_18918831_A/Barnacle_Goose/Netherlands/24000825−002/2024_H5N1_2024−01−11
EPI_ISL_11112346_A/Barnacle_Goose/Netherlands/13/2022_H5N1_2022−02−23
EPI_ISL_10993074_A/Mallard/Netherlands/2/2022_H5N1_2022−02−14
EPI_ISL_7267253_A/gull/Netherlands/21039180−002/2021_H5N1_2021−11−18
EPI_ISL_10993075_A/Mallard/Netherlands/4/2021_H5N1_2021−11−29
Root
0.001
Season 2020-2021
Season 2022-2023
Peregrine Falcon
Season 2021-2022
Season 2023-2024

NA_N8

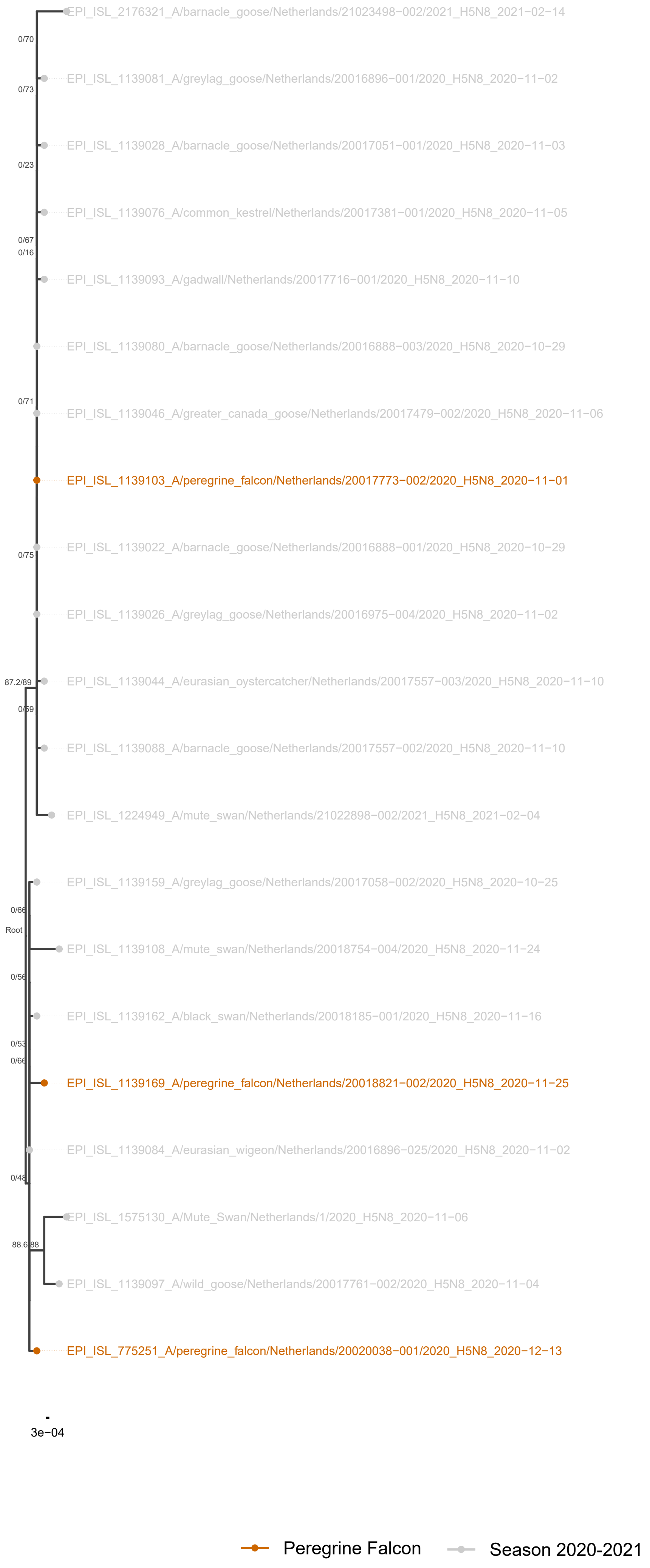
EPI_ISL_2176321_A/barnacle_goose/Netherlands/21023498−002/2021_H5N8_2021−02−14
EPI_ISL_1139081_A/greylag_goose/Netherlands/20016896−001/2020_H5N8_2020−11−02
EPI_ISL_1139028_A/barnacle_goose/Netherlands/20017051−001/2020_H5N8_2020−11−03
EPI_ISL_1139076_A/common_kestrel/Netherlands/20017381−001/2020_H5N8_2020−11−05
EPI_ISL_1139093_A/gadwall/Netherlands/20017716−001/2020_H5N8_2020−11−10
EPI_ISL_1139080_A/barnacle_goose/Netherlands/20016888−003/2020_H5N8_2020−10−29
EPI_ISL_1139046_A/greater_canada_goose/Netherlands/20017479−002/2020_H5N8_2020−11−06
EPI_ISL_1139103_A/peregrine_falcon/Netherlands/20017773−002/2020_H5N8_2020−11−01
EPI_ISL_1139022_A/barnacle_goose/Netherlands/20016888−001/2020_H5N8_2020−10−29
EPI_ISL_1139026_A/greylag_goose/Netherlands/20016975−004/2020_H5N8_2020−11−02
EPI_ISL_1139044_A/eurasian_oystercatcher/Netherlands/20017557−003/2020_H5N8_2020−11−10
EPI_ISL_1139088_A/barnacle_goose/Netherlands/20017557−002/2020_H5N8_2020−11−10
EPI_ISL_1224949_A/mute_swan/Netherlands/21022898−002/2021_H5N8_2021−02−04
EPI_ISL_1139159_A/greylag_goose/Netherlands/20017058−002/2020_H5N8_2020−10−25
EPI_ISL_1139108_A/mute_swan/Netherlands/20018754−004/2020_H5N8_2020−11−24
EPI_ISL_1139162_A/black_swan/Netherlands/20018185−001/2020_H5N8_2020−11−16
EPI_ISL_1139169_A/peregrine_falcon/Netherlands/20018821−002/2020_H5N8_2020−11−25
EPI_ISL_1139084_A/eurasian_wigeon/Netherlands/20016896−025/2020_H5N8_2020−11−02
EPI_ISL_1575130_A/Mute_Swan/Netherlands/1/2020_H5N8_2020−11−06
EPI_ISL_1139097_A/wild_goose/Netherlands/20017761−002/2020_H5N8_2020−11−04
EPI_ISL_775251_A/peregrine_falcon/Netherlands/20020038−001/2020_H5N8_2020−12−13
Root
3e−04
Peregrine Falcon
Season 2020-2021

MP

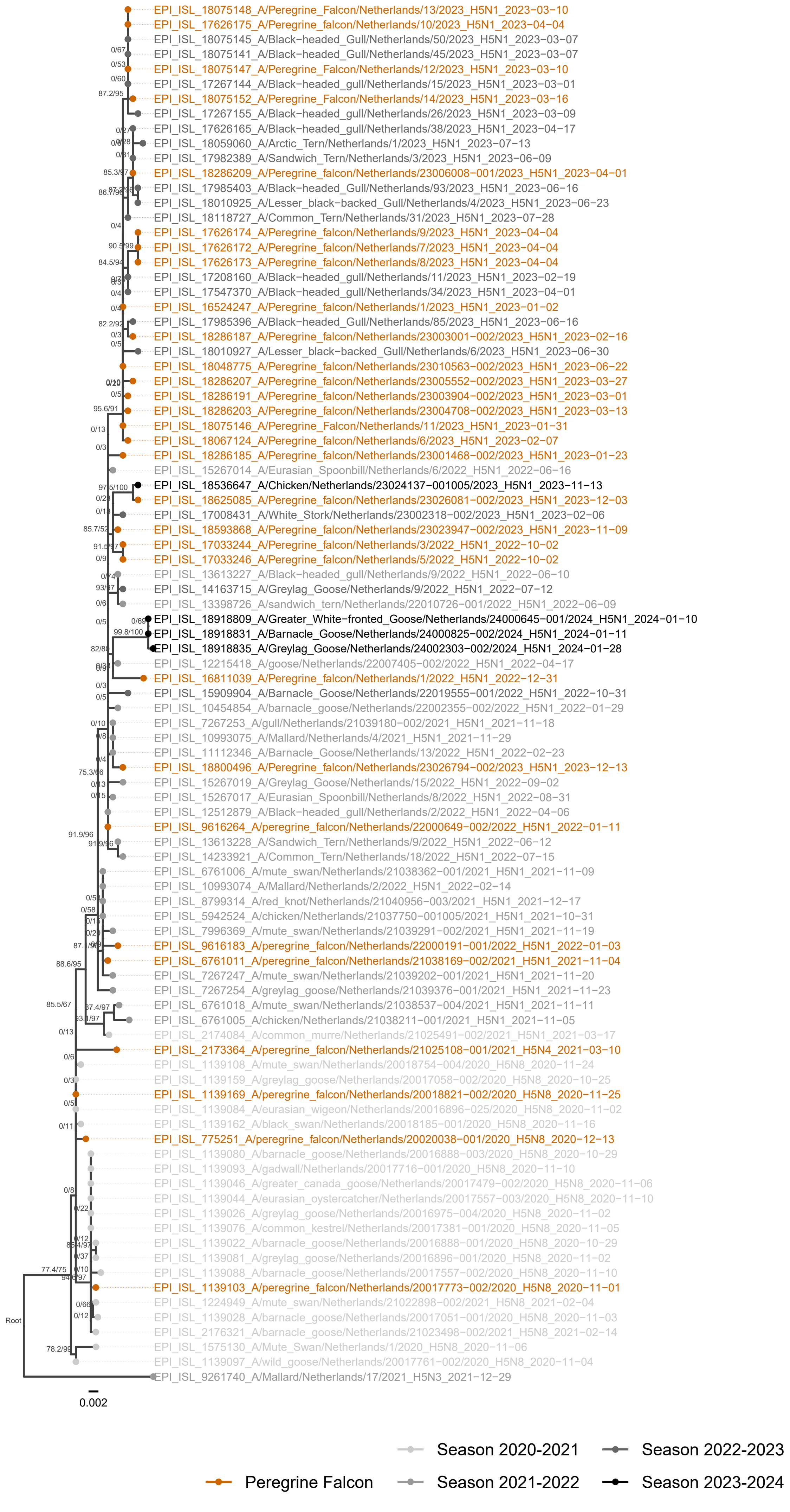

EPI_ISL_18075148_A/Peregrine_Falcon/Netherlands/13/2023_H5N1_2023−03−10
EPI_ISL_17626175_A/Peregrine_falcon/Netherlands/10/2023_H5N1_2023−04−04
EPI_ISL_18075145_A/Black−headed_Gull/Netherlands/50/2023_H5N1_2023−03−07
EPI_ISL_18075141_A/Black−headed_Gull/Netherlands/45/2023_H5N1_2023−03−07
EPI_ISL_18075147_A/Peregrine_Falcon/Netherlands/12/2023_H5N1_2023−03−10
EPI_ISL_17267144_A/Black−headed_gull/Netherlands/15/2023_H5N1_2023−03−01
EPI_ISL_18075152_A/Peregrine_Falcon/Netherlands/14/2023_H5N1_2023−03−16
EPI_ISL_17267155_A/Black−headed_gull/Netherlands/26/2023_H5N1_2023−03−09
EPI_ISL_17626165_A/Black−headed_gull/Netherlands/38/2023_H5N1_2023−04−17
EPI_ISL_18059060_A/Arctic_Tern/Netherlands/1/2023_H5N1_2023−07−13
EPI_ISL_17982389_A/Sandwich_Tern/Netherlands/3/2023_H5N1_2023−06−09
EPI_ISL_18286209_A/Peregrine_falcon/Netherlands/23006008−001/2023_H5N1_2023−04−01
EPI_ISL_17985403_A/Black−headed_Gull/Netherlands/93/2023_H5N1_2023−06−16
EPI_ISL_18010925_A/Lesser_black−backed_Gull/Netherlands/4/2023_H5N1_2023−06−23
EPI_ISL_18118727_A/Common_Tern/Netherlands/31/2023_H5N1_2023−07−28
EPI_ISL_17626174_A/Peregrine_falcon/Netherlands/9/2023_H5N1_2023−04−04
EPI_ISL_17626172_A/Peregrine_falcon/Netherlands/7/2023_H5N1_2023−04−04
EPI_ISL_17626173_A/Peregrine_falcon/Netherlands/8/2023_H5N1_2023−04−04
EPI_ISL_17208160_A/Black−headed_gull/Netherlands/11/2023_H5N1_2023−02−19
EPI_ISL_17547370_A/Black−headed_gull/Netherlands/34/2023_H5N1_2023−04−01
EPI_ISL_16524247_A/Peregrine_falcon/Netherlands/1/2023_H5N1_2023−01−02
EPI_ISL_17985396_A/Black−headed_Gull/Netherlands/85/2023_H5N1_2023−06−16
EPI_ISL_18286187_A/Peregrine_falcon/Netherlands/23003001−002/2023_H5N1_2023−02−16
EPI_ISL_18010927_A/Lesser_black−backed_Gull/Netherlands/6/2023_H5N1_2023−06−30
EPI_ISL_18048775_A/Peregrine_falcon/Netherlands/23010563−002/2023_H5N1_2023−06−22
EPI_ISL_18286207_A/Peregrine_falcon/Netherlands/23005552−002/2023_H5N1_2023−03−27
EPI_ISL_18286191_A/Peregrine_falcon/Netherlands/23003904−002/2023_H5N1_2023−03−01
EPI_ISL_18286203_A/Peregrine_falcon/Netherlands/23004708−002/2023_H5N1_2023−03−13
EPI_ISL_18075146_A/Peregrine_Falcon/Netherlands/11/2023_H5N1_2023−01−31
EPI_ISL_18067124_A/Peregrine_falcon/Netherlands/6/2023_H5N1_2023−02−07
EPI_ISL_18286185_A/Peregrine_falcon/Netherlands/23001468−002/2023_H5N1_2023−01−23
EPI_ISL_15267014_A/Eurasian_Spoonbill/Netherlands/6/2022_H5N1_2022−06−16
EPI_ISL_18536647_A/Chicken/Netherlands/23024137−001005/2023_H5N1_2023−11−13
EPI_ISL_18625085_A/Peregrine_falcon/Netherlands/23026081−002/2023_H5N1_2023−12−03
EPI_ISL_17008431_A/White_Stork/Netherlands/23002318−002/2023_H5N1_2023−02−06
EPI_ISL_18593868_A/Peregrine_falcon/Netherlands/23023947−002/2023_H5N1_2023−11−09
EPI_ISL_17033244_A/Peregrine_falcon/Netherlands/3/2022_H5N1_2022−10−02
EPI_ISL_17033246_A/Peregrine_falcon/Netherlands/5/2022_H5N1_2022−10−02
EPI_ISL_13613227_A/Black−headed_gull/Netherlands/9/2022_H5N1_2022−06−10
EPI_ISL_14163715_A/Greylag_Goose/Netherlands/9/2022_H5N1_2022−07−12
EPI_ISL_13398726_A/sandwich_tern/Netherlands/22010726−001/2022_H5N1_2022−06−09
EPI_ISL_18918809_A/Greater_White−fronted_Goose/Netherlands/24000645−001/2024_H5N1_2024−01−10
EPI_ISL_18918831_A/Barnacle_Goose/Netherlands/24000825−002/2024_H5N1_2024−01−11
EPI_ISL_18918835_A/Greylag_Goose/Netherlands/24002303−002/2024_H5N1_2024−01−28
EPI_ISL_12215418_A/goose/Netherlands/22007405−002/2022_H5N1_2022−04−17
EPI_ISL_16811039_A/Peregrine_falcon/Netherlands/1/2022_H5N1_2022−12−31
EPI_ISL_15909904_A/Barnacle_Goose/Netherlands/22019555−001/2022_H5N1_2022−10−31
EPI_ISL_10454854_A/barnacle_goose/Netherlands/22002355−002/2022_H5N1_2022−01−29
EPI_ISL_7267253_A/gull/Netherlands/21039180−002/2021_H5N1_2021−11−18
EPI_ISL_10993075_A/Mallard/Netherlands/4/2021_H5N1_2021−11−29
EPI_ISL_11112346_A/Barnacle_Goose/Netherlands/13/2022_H5N1_2022−02−23
EPI_ISL_18800496_A/Peregrine_falcon/Netherlands/23026794−002/2023_H5N1_2023−12−13
EPI_ISL_15267019_A/Greylag_Goose/Netherlands/15/2022_H5N1_2022−09−02
EPI_ISL_15267017_A/Eurasian_Spoonbill/Netherlands/8/2022_H5N1_2022−08−31
EPI_ISL_12512879_A/Black−headed_gull/Netherlands/2/2022_H5N1_2022−04−06
EPI_ISL_9616264_A/peregrine_falcon/Netherlands/22000649−002/2022_H5N1_2022−01−11
EPI_ISL_13613228_A/Sandwich_Tern/Netherlands/9/2022_H5N1_2022−06−12
EPI_ISL_14233921_A/Common_Tern/Netherlands/18/2022_H5N1_2022−07−15
EPI_ISL_6761006_A/mute_swan/Netherlands/21038362−001/2021_H5N1_2021−11−09
EPI_ISL_10993074_A/Mallard/Netherlands/2/2022_H5N1_2022−02−14
EPI_ISL_8799314_A/red_knot/Netherlands/21040956−003/2021_H5N1_2021−12−17
EPI_ISL_5942524_A/chicken/Netherlands/21037750−001005/2021_H5N1_2021−10−31
EPI_ISL_7996369_A/mute_swan/Netherlands/21039291−002/2021_H5N1_2021−11−19
EPI_ISL_9616183_A/peregrine_falcon/Netherlands/22000191−001/2022_H5N1_2022−01−03
EPI_ISL_6761011_A/peregrine_falcon/Netherlands/21038169−002/2021_H5N1_2021−11−04
EPI_ISL_7267247_A/mute_swan/Netherlands/21039202−001/2021_H5N1_2021−11−20
EPI_ISL_7267254_A/greylag_goose/Netherlands/21039376−001/2021_H5N1_2021−11−23
EPI_ISL_6761018_A/mute_swan/Netherlands/21038537−004/2021_H5N1_2021−11−11
EPI_ISL_6761005_A/chicken/Netherlands/21038211−001/2021_H5N1_2021−11−05
EPI_ISL_2174084_A/common_murre/Netherlands/21025491−002/2021_H5N1_2021−03−17
EPI_ISL_2173364_A/peregrine_falcon/Netherlands/21025108−001/2021_H5N4_2021−03−10
EPI_ISL_1139108_A/mute_swan/Netherlands/20018754−004/2020_H5N8_2020−11−24
EPI_ISL_1139159_A/greylag_goose/Netherlands/20017058−002/2020_H5N8_2020−10−25
EPI_ISL_1139169_A/peregrine_falcon/Netherlands/20018821−002/2020_H5N8_2020−11−25
EPI_ISL_1139084_A/eurasian_wigeon/Netherlands/20016896−025/2020_H5N8_2020−11−02
EPI_ISL_1139162_A/black_swan/Netherlands/20018185−001/2020_H5N8_2020−11−16
EPI_ISL_775251_A/peregrine_falcon/Netherlands/20020038−001/2020_H5N8_2020−12−13
EPI_ISL_1139080_A/barnacle_goose/Netherlands/20016888−003/2020_H5N8_2020−10−29
EPI_ISL_1139093_A/gadwall/Netherlands/20017716−001/2020_H5N8_2020−11−10
EPI_ISL_1139046_A/greater_canada_goose/Netherlands/20017479−002/2020_H5N8_2020−11−06
EPI_ISL_1139044_A/eurasian_oystercatcher/Netherlands/20017557−003/2020_H5N8_2020−11−10
EPI_ISL_1139026_A/greylag_goose/Netherlands/20016975−004/2020_H5N8_2020−11−02
EPI_ISL_1139076_A/common_kestrel/Netherlands/20017381−001/2020_H5N8_2020−11−05
EPI_ISL_1139022_A/barnacle_goose/Netherlands/20016888−001/2020_H5N8_2020−10−29
EPI_ISL_1139081_A/greylag_goose/Netherlands/20016896−001/2020_H5N8_2020−11−02
EPI_ISL_1139088_A/barnacle_goose/Netherlands/20017557−002/2020_H5N8_2020−11−10
EPI_ISL_1139103_A/peregrine_falcon/Netherlands/20017773−002/2020_H5N8_2020−11−01
EPI_ISL_1224949_A/mute_swan/Netherlands/21022898−002/2021_H5N8_2021−02−04
EPI_ISL_1139028_A/barnacle_goose/Netherlands/20017051−001/2020_H5N8_2020−11−03
EPI_ISL_2176321_A/barnacle_goose/Netherlands/21023498−002/2021_H5N8_2021−02−14
EPI_ISL_1575130_A/Mute_Swan/Netherlands/1/2020_H5N8_2020−11−06
EPI_ISL_1139097_A/wild_goose/Netherlands/20017761−002/2020_H5N8_2020−11−04
EPI_ISL_9261740_A/Mallard/Netherlands/17/2021_H5N3_2021−12−29
Root
0.002
Season 2020-2021
Season 2022-2023
Peregrine Falcon
Season 2021-2022
Season 2023-2024

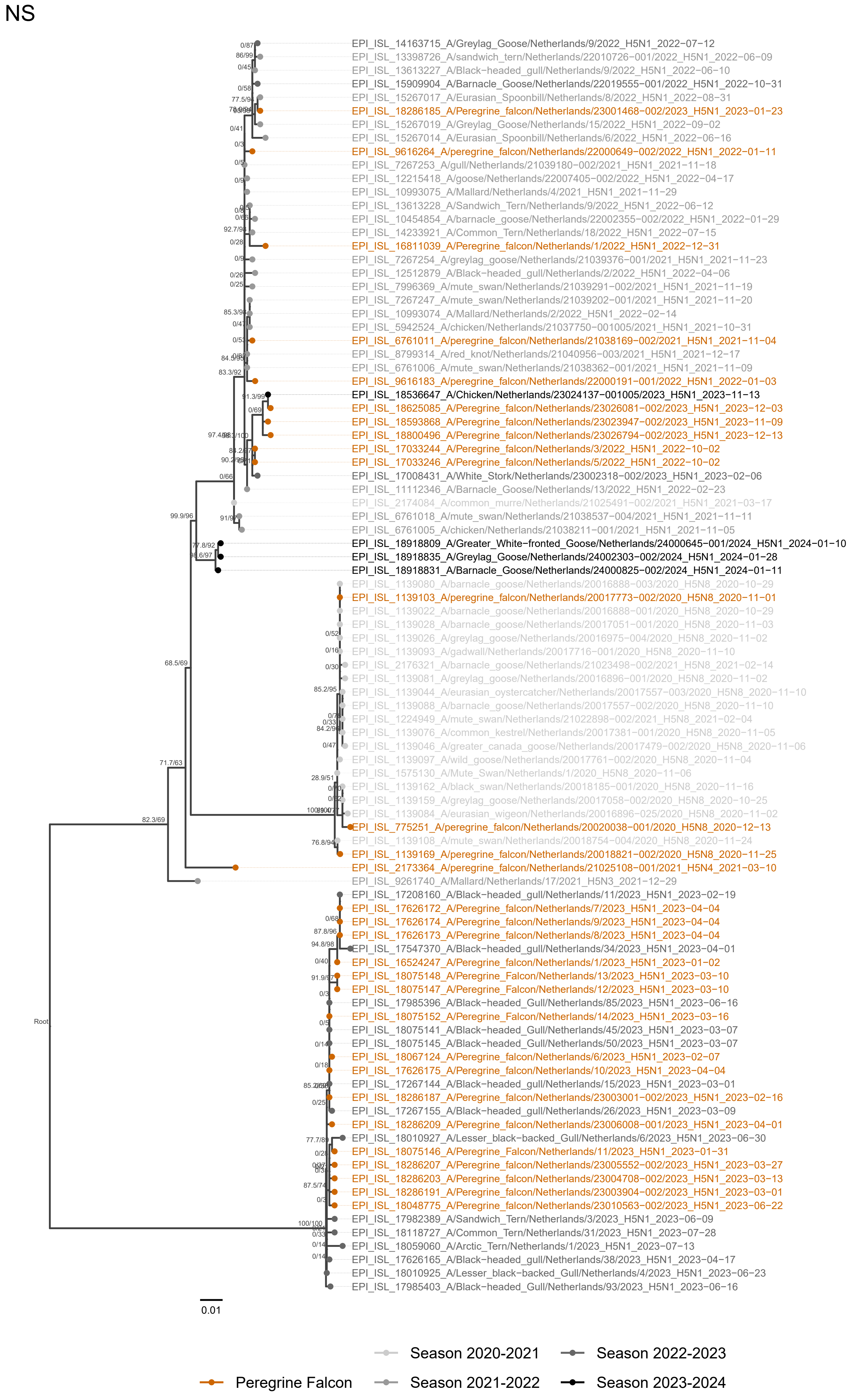
NS
EPI_ISL_14163715_A/Greylag_Goose/Netherlands/9/2022_H5N1_2022−07−12
EPI_ISL_13398726_A/sandwich_tern/Netherlands/22010726−001/2022_H5N1_2022−06−09
EPI_ISL_13613227_A/Black−headed_gull/Netherlands/9/2022_H5N1_2022−06−10
EPI_ISL_15909904_A/Barnacle_Goose/Netherlands/22019555−001/2022_H5N1_2022−10−31
EPI_ISL_15267017_A/Eurasian_Spoonbill/Netherlands/8/2022_H5N1_2022−08−31
EPI_ISL_18286185_A/Peregrine_falcon/Netherlands/23001468−002/2023_H5N1_2023−01−23
EPI_ISL_15267019_A/Greylag_Goose/Netherlands/15/2022_H5N1_2022−09−02
EPI_ISL_15267014_A/Eurasian_Spoonbill/Netherlands/6/2022_H5N1_2022−06−16
EPI_ISL_9616264_A/peregrine_falcon/Netherlands/22000649−002/2022_H5N1_2022−01−11
EPI_ISL_7267253_A/gull/Netherlands/21039180−002/2021_H5N1_2021−11−18
EPI_ISL_12215418_A/goose/Netherlands/22007405−002/2022_H5N1_2022−04−17
EPI_ISL_10993075_A/Mallard/Netherlands/4/2021_H5N1_2021−11−29
EPI_ISL_13613228_A/Sandwich_Tern/Netherlands/9/2022_H5N1_2022−06−12
EPI_ISL_10454854_A/barnacle_goose/Netherlands/22002355−002/2022_H5N1_2022−01−29
EPI_ISL_14233921_A/Common_Tern/Netherlands/18/2022_H5N1_2022−07−15
EPI_ISL_16811039_A/Peregrine_falcon/Netherlands/1/2022_H5N1_2022−12−31
EPI_ISL_7267254_A/greylag_goose/Netherlands/21039376−001/2021_H5N1_2021−11−23
EPI_ISL_12512879_A/Black−headed_gull/Netherlands/2/2022_H5N1_2022−04−06
EPI_ISL_7996369_A/mute_swan/Netherlands/21039291−002/2021_H5N1_2021−11−19
EPI_ISL_7267247_A/mute_swan/Netherlands/21039202−001/2021_H5N1_2021−11−20
EPI_ISL_10993074_A/Mallard/Netherlands/2/2022_H5N1_2022−02−14
EPI_ISL_5942524_A/chicken/Netherlands/21037750−001005/2021_H5N1_2021−10−31
EPI_ISL_6761011_A/peregrine_falcon/Netherlands/21038169−002/2021_H5N1_2021−11−04
EPI_ISL_8799314_A/red_knot/Netherlands/21040956−003/2021_H5N1_2021−12−17
EPI_ISL_6761006_A/mute_swan/Netherlands/21038362−001/2021_H5N1_2021−11−09
EPI_ISL_9616183_A/peregrine_falcon/Netherlands/22000191−001/2022_H5N1_2022−01−03
EPI_ISL_18536647_A/Chicken/Netherlands/23024137−001005/2023_H5N1_2023−11−13
EPI_ISL_18625085_A/Peregrine_falcon/Netherlands/23026081−002/2023_H5N1_2023−12−03
EPI_ISL_18593868_A/Peregrine_falcon/Netherlands/23023947−002/2023_H5N1_2023−11−09
EPI_ISL_18800496_A/Peregrine_falcon/Netherlands/23026794−002/2023_H5N1_2023−12−13
EPI_ISL_17033244_A/Peregrine_falcon/Netherlands/3/2022_H5N1_2022−10−02
EPI_ISL_17033246_A/Peregrine_falcon/Netherlands/5/2022_H5N1_2022−10−02
EPI_ISL_17008431_A/White_Stork/Netherlands/23002318−002/2023_H5N1_2023−02−06
EPI_ISL_11112346_A/Barnacle_Goose/Netherlands/13/2022_H5N1_2022−02−23
EPI_ISL_2174084_A/common_murre/Netherlands/21025491−002/2021_H5N1_2021−03−17
EPI_ISL_6761018_A/mute_swan/Netherlands/21038537−004/2021_H5N1_2021−11−11
EPI_ISL_6761005_A/chicken/Netherlands/21038211−001/2021_H5N1_2021−11−05
EPI_ISL_18918809_A/Greater_White−fronted_Goose/Netherlands/24000645−001/2024_H5N1_2024−01−10
EPI_ISL_18918835_A/Greylag_Goose/Netherlands/24002303−002/2024_H5N1_2024−01−28
EPI_ISL_18918831_A/Barnacle_Goose/Netherlands/24000825−002/2024_H5N1_2024−01−11
EPI_ISL_1139080_A/barnacle_goose/Netherlands/20016888−003/2020_H5N8_2020−10−29
EPI_ISL_1139103_A/peregrine_falcon/Netherlands/20017773−002/2020_H5N8_2020−11−01
EPI_ISL_1139022_A/barnacle_goose/Netherlands/20016888−001/2020_H5N8_2020−10−29
EPI_ISL_1139028_A/barnacle_goose/Netherlands/20017051−001/2020_H5N8_2020−11−03
EPI_ISL_1139026_A/greylag_goose/Netherlands/20016975−004/2020_H5N8_2020−11−02
EPI_ISL_1139093_A/gadwall/Netherlands/20017716−001/2020_H5N8_2020−11−10
EPI_ISL_2176321_A/barnacle_goose/Netherlands/21023498−002/2021_H5N8_2021−02−14
EPI_ISL_1139081_A/greylag_goose/Netherlands/20016896−001/2020_H5N8_2020−11−02
EPI_ISL_1139044_A/eurasian_oystercatcher/Netherlands/20017557−003/2020_H5N8_2020−11−10
EPI_ISL_1139088_A/barnacle_goose/Netherlands/20017557−002/2020_H5N8_2020−11−10
EPI_ISL_1224949_A/mute_swan/Netherlands/21022898−002/2021_H5N8_2021−02−04
EPI_ISL_1139076_A/common_kestrel/Netherlands/20017381−001/2020_H5N8_2020−11−05
EPI_ISL_1139046_A/greater_canada_goose/Netherlands/20017479−002/2020_H5N8_2020−11−06
EPI_ISL_1139097_A/wild_goose/Netherlands/20017761−002/2020_H5N8_2020−11−04
EPI_ISL_1575130_A/Mute_Swan/Netherlands/1/2020_H5N8_2020−11−06
EPI_ISL_1139162_A/black_swan/Netherlands/20018185−001/2020_H5N8_2020−11−16
EPI_ISL_1139159_A/greylag_goose/Netherlands/20017058−002/2020_H5N8_2020−10−25
EPI_ISL_1139084_A/eurasian_wigeon/Netherlands/20016896−025/2020_H5N8_2020−11−02
EPI_ISL_775251_A/peregrine_falcon/Netherlands/20020038−001/2020_H5N8_2020−12−13
EPI_ISL_1139108_A/mute_swan/Netherlands/20018754−004/2020_H5N8_2020−11−24
EPI_ISL_1139169_A/peregrine_falcon/Netherlands/20018821−002/2020_H5N8_2020−11−25
EPI_ISL_2173364_A/peregrine_falcon/Netherlands/21025108−001/2021_H5N4_2021−03−10
EPI_ISL_9261740_A/Mallard/Netherlands/17/2021_H5N3_2021−12−29
EPI_ISL_17208160_A/Black−headed_gull/Netherlands/11/2023_H5N1_2023−02−19
EPI_ISL_17626172_A/Peregrine_falcon/Netherlands/7/2023_H5N1_2023−04−04
EPI_ISL_17626174_A/Peregrine_falcon/Netherlands/9/2023_H5N1_2023−04−04
EPI_ISL_17626173_A/Peregrine_falcon/Netherlands/8/2023_H5N1_2023−04−04
EPI_ISL_17547370_A/Black−headed_gull/Netherlands/34/2023_H5N1_2023−04−01
EPI_ISL_16524247_A/Peregrine_falcon/Netherlands/1/2023_H5N1_2023−01−02
EPI_ISL_18075148_A/Peregrine_Falcon/Netherlands/13/2023_H5N1_2023−03−10
EPI_ISL_18075147_A/Peregrine_Falcon/Netherlands/12/2023_H5N1_2023−03−10
EPI_ISL_17985396_A/Black−headed_Gull/Netherlands/85/2023_H5N1_2023−06−16
EPI_ISL_18075152_A/Peregrine_Falcon/Netherlands/14/2023_H5N1_2023−03−16
EPI_ISL_18075141_A/Black−headed_Gull/Netherlands/45/2023_H5N1_2023−03−07
EPI_ISL_18075145_A/Black−headed_Gull/Netherlands/50/2023_H5N1_2023−03−07
EPI_ISL_18067124_A/Peregrine_falcon/Netherlands/6/2023_H5N1_2023−02−07
EPI_ISL_17626175_A/Peregrine_falcon/Netherlands/10/2023_H5N1_2023−04−04
EPI_ISL_17267144_A/Black−headed_gull/Netherlands/15/2023_H5N1_2023−03−01
EPI_ISL_18286187_A/Peregrine_falcon/Netherlands/23003001−002/2023_H5N1_2023−02−16
EPI_ISL_17267155_A/Black−headed_gull/Netherlands/26/2023_H5N1_2023−03−09
EPI_ISL_18286209_A/Peregrine_falcon/Netherlands/23006008−001/2023_H5N1_2023−04−01
EPI_ISL_18010927_A/Lesser_black−backed_Gull/Netherlands/6/2023_H5N1_2023−06−30
EPI_ISL_18075146_A/Peregrine_Falcon/Netherlands/11/2023_H5N1_2023−01−31
EPI_ISL_18286207_A/Peregrine_falcon/Netherlands/23005552−002/2023_H5N1_2023−03−27
EPI_ISL_18286203_A/Peregrine_falcon/Netherlands/23004708−002/2023_H5N1_2023−03−13
EPI_ISL_18286191_A/Peregrine_falcon/Netherlands/23003904−002/2023_H5N1_2023−03−01
EPI_ISL_18048775_A/Peregrine_falcon/Netherlands/23010563−002/2023_H5N1_2023−06−22
EPI_ISL_17982389_A/Sandwich_Tern/Netherlands/3/2023_H5N1_2023−06−09
EPI_ISL_18118727_A/Common_Tern/Netherlands/31/2023_H5N1_2023−07−28
EPI_ISL_18059060_A/Arctic_Tern/Netherlands/1/2023_H5N1_2023−07−13
EPI_ISL_17626165_A/Black−headed_gull/Netherlands/38/2023_H5N1_2023−04−17
EPI_ISL_18010925_A/Lesser_black−backed_Gull/Netherlands/4/2023_H5N1_2023−06−23
EPI_ISL_17985403_A/Black−headed_Gull/Netherlands/93/2023_H5N1_2023−06−16
Root
0.01
Season 2020-2021
Season 2022-2023
Peregrine Falcon
Season 2021-2022
Season 2023-2024
